# Supplementary material for: WordCommentsAnalyzer: A windows software tool for qualitative research
Source: F1000Res. 2018 Sep 4;7:536. Originally published 2018 May 3. [Version 2] doi: 10.12688/f1000research.14819.2 (PMC6137411; doi:10.12688/f1000research.14819.2)
Supplement: Supplementary file 3 [file f1000research-7-17614-s0002.tgz › b2a79496-4ada-47c4-9682-a99ce867836d.zip!/Eight Iranian Health Journals/The Journal of Toloo-e-Behdasht_15.docx]

© The Journal of Toloo-e-Behdasht. This work is licensed under CC BY 4.0 (https://creativecommons.org/licenses/by/4.0/).

Volume & Issue: دوره 14 - شماره 6

Title: بررسی نقاط حادثه خیز جاده ای و ارتباط آن با متغیرهای محیطی و دموگرافیک با استفاده از روشHot Zone در محور یزد-کرمان

http://tbj.ssu.ac.ir/article-1-1996-fa.html

مقدمه: امروزه در کشورهای مختلف با اجرای مدلهای متفاوت در نرم افزار سیستم اطلاعات جغرافیایی، سعی در شناسایی مکانهای خطرناک شبکه های راه می کنند، هدف ما نیز شناسایی موقعیت های خطرناک بر روی قسمتی از بزرگراه یزد- کرمان به طول 212 کیلومتر در حوزه ی استحفاظی استان یزد، با استفاده از روش Hot Zone در نرم افزار GIS و ارتباط آن با متغییرهای محیطی و دموگرافیک بوده است. روش بررسی:با استفاده از روش Hot Zone بر اساس آستانه های تکرار تصادفات و شدت تصادفات در هر یک کیلومتر از جاده  به عنوان یک واحد فضایی پایه(BSU)،مکانهای خطرناک بزرگراه که در 2 تعریف Hot Zone،Yellow Zone  (H-Y Zones) گنجانده شده اند به تفکیک دو مسیر رفت و برگشت شناسایی شدند. سپس متغییرهای ساعت تصادفات، شرایط جوی، نوع وسیله ی نقلیه، علت تصادفات، سن و جنس و وضعیت سلامت رانندگان مورد بررسی قرار گرفتند. یافته ها: بر اساس نتایج بدست آمده 17% از کل مسیر 212 کیلومتری بزرگراه مورد مطالعه به عنوان موقعیتهای خطرناک شناسایی شدند. بیشترین تصادفات (34%) در نقاط حادثه خیز مربوط به رانندگان گروه سنی 40-31 سال می باشد. بر اساس آزمون T-Test، میانگین ساعت وقوع تصادفات در دو مسیر با یکدیگر معنی دار شدند (p<0/05). وسایل نقلیه از نوع سواری، تریلی و کامیون در وقوع این تصادفات در هر دو مسیر، به ترتیب بالاترین درصد فراوانی را به خود اختصاص دادند و بیشترین علت تصادفات، عدم توجه به جلو و عدم کنترل وسیله نقلیه می باشد. همچنین بیشترین تصادفات در شرایط آب و هوایی آفتابی اتفاق افتاده اند. نتیجه گیری:با توجه به اینکه تعداد موقعیتهای خطرناک در نزدیک خروجی و ورودی به شهر، جاده های فرعی وارد شده و جدا شده از بزرگراه و تقاطع ها بیشتر می شود، به نظر می رسد توجه به اصول طراحی مناسب در شکل هندسی جاده باعث کاهش چشمگیر تصادفات در این مکانها شود. همچنین طراحی مناسب جاده نقش موثری در کاهش تاثیر عوامل محیطی چون شرایط جوی، ساعات وقوع تصادفات و حتی نوع وسیله نقلیه در رخداد تصادفات خواهند داشت.ضمناًتوجه به آموزش رانندگان علی الخصوص گروه سنی 40–21 سال، کمک شایانی در به حداقل رساندن نقش عوامل انسانی در بروز تصادفات و صیانت از نیروی کار خواهد داشت.

Title: حذف رنگ آزو از فاضلاب مصنوعی با استفاده ازنانو-دیاتومیت تثبیت شده در آلژینات کلسیم

http://tbj.ssu.ac.ir/article-1-2000-fa.html

مقدمه:حضور رنگ های آلی تخلیه شده در محیط های آبی توسط صنایع نساجی می تواند موجب اثرات نامطلوب بر زندگی آبزیان و در نتیجه سلامت انسان باشد. بنابراین، رنگ زدایی از محیط های آبی برای حفاظت از محیط زیست ضروری می باشد. به همین دلیل، در این مطالعه نانو ذرات دیاتومیت در آلژینات کلسیم تثبیت شده و به عنوان جاذب نانو کامپوزیت برای حذف رنگ آلی آزویی (دایرکت آبی15) از محیط های آبی مورد استفاده واقع شد. روش بررسی: در ابتدا، دیاتومیت ایرانی داخل یک آسیاب سیاره ای مجهز به کاربید تنگستن طی مدت 20 ساعت خرد شده و به اندازه نانو تبدیل گردید. برای تثبیت نانو ذرات دیاتومیت، محلول آلژینات سدیم 2 درصد استفاده شد. میکروسکوپ الکترونی روبشی، پراش اشعه ایکس و آنالیز اسپکتروفتومتر مادون قرمز، برای تعیین مشخصات نانو ذرات دیاتومیت تثبیت شده استفاده گردید. ارلن مایر های 50 میلی لیتری بعنوان راکتورهای آزمایشگاهی جریان ناپیوسته استفاده شدند. غلظت های مورد نظر محلول رنگ، بوسیله رقیق سازی محلول ذخیره (یک گرم بر لیتر) آماده شدند. تأثیر پارامترهای مختلف عملیاتی نظیر pH، زمان تماس، مقدار جاذب، غلظت اولیه رنگ به همراه ایزوترم و کینتیک جذب مورد بررسی قرار گرفتند. پس از هر آزمایش میزان غلظت رنگ باقیمانده به روش اسپکتروفتومتری اندازه گیری گردید. یافته ها:مطابق با نتایج، با افزایش زمان تماس و دوزاژ جاذب، میزان جذب رنگ آلی افزایش یافت، در حالی که کاهش غلظت اولیه رنگ منجر به کاهش میزان جذب آن گردید. جذب رنگ دایرکت آبی15 در شرایط قلیایی مطلوب تر بود. دیاتومیت تثبیت شده در مقایسه با دیاتومیت خام، منجر به افزایش جذب رنگ گردید. مطابق با ضریب همبستگی بدست آمده، جذب دایرکت آبی15 از مدل کینتیک درجه دوم کاذب و مدل ایزوترم لانگموئر تبعیت نمود. حداکثر ظرفیت جذب نانوکامپوزیت دیاتومیت/آلژینات برای دایرکت آبی15 معادل 22/33  میلی گرم بر گرم  بدست آمد.  نتیجه گیری:نتایج این تحقیق نشان داد که نانوکامپوزیت دیاتومیت/آلژینات، به دلیل کارایی بالا، دردسترس بودن معادن دیاتومیت در کشور و هزینه پایین، می تواند به طور مؤثری برای رنگ زدایی ازپساب های نساجی به کار رود.

Title: بررسی رابطه بین آگاهی از علم ارگونومی و میزان آسیب های شغلی کادر پرستاری در بیمارستان های آموزشی منتخب دانشگاه علوم پزشکی شهید صدوقی یزد

http://tbj.ssu.ac.ir/article-1-2003-fa.html

مقدمه: ارگونومی به معنای قاعده و قانون کار می باشد و به بررسی روابط انسان با محیط کارش با محور بهبود شرایط زندگی و کاری فرد می پردازد. لذا مطالعه ی حاضر قصد دارد با تعیین میزان آگاهی از اصول ارگونومی و شرایط کاری پرسنل، زمینه را برای ارائه ی راهکارهای کنترلی مناسب در برابر آسیب های شغلی و اختلالات اسکلتی عضلانی در بیمارستان ها فراهم نماید. روش بررسی: در این مطالعه مقطعی تعداد 523 نفر از پرسنل کادر پرستاری در 4 بیمارستان شهر یزد مورد بررسی قرار گرفتند. ابزار جمع آوری اطلاعات پرسشنامه بود. متغیرهای اصلی مورد بررسی میزان آگاهی از اصول ارگونومی، شرایط کاری، میزان آسیب های شغلی و متغیرهای دموگرافیک بودند. اطلاعات پس از استخراج، وارد نرم افزار SPSS نسخه 16 گردیده و با استفاده از آزمون های آماری توصیفی، کلموگروف اسمیرنوف، کای‌دو، t-test مستقل، من ویتنی و پیرسون مورد پردازش قرار گرفت. یافته ها: میزان آگاهی پرستاران در زمینه ی ارگونومی با میانگین) 076±2.86 (از 5 امتیاز در حد متوسط، شرایط کاری پرستاران با میانگین )0.58±2.41 (در حد ضعیف و میزان آسیب های شغلی از قبیل اختلالات اسکلتی عضلانی با میانگین )0.87±2.67( نیز در حد ضعیف بود. بین میزان آگاهی از علم ارگونومی و میزان آسیب های شغلی از لحاظ آماری ارتباط معکوس(P-value=0.04) و بین شرایط کاری و میزان آسیب های شغلی، ارتباط مستقیم معنی داری مشاهده گردید (P-value<0.001). علت اصلی بروز اختلالات و آسیب های شغلی در پرستاران مورد مطالعه انجام فعالیت به صورت ایستاده بود. همچنین بین میزان تحصیلات افراد با مشکلات و اختلالات اسکلتی عضلانی ارتباط معنی داری مشاهده گردید (P-value<0.001). نتیجه­گیری: برنامه های آموزشی مدون و جامع و به کارگیری مداخلات ارگونومیک جهت بهبود شرایط کاری می تواند نقش موثری در کاهش آسیب های شغلی پرستاران داشته باشد.

Title: استرس شغلی و سابقه بیماریها در رانندگان نوبت کار و غیر نوبت کار آژانس های درون شهری

http://tbj.ssu.ac.ir/article-1-2004-fa.html

مقدمه : یکی از معضلات اساسی در جوامع صنعتی، استرس شغلی می باشد.  لذا این مطالعه با هدف بررسی استرس شغلی و سابقه بیماری ها در رانندگان آژانس های درون شهری یزد در دو گروه نوبت کار و غیر نوبت کار انجام گردید. روش­­ ­بررسی : مطالعه حاضر یک مطالعه پژوهشی تحلیلی است که به روش موردی- شاهدی در سال 1392 و در رانندگان آژانس های درون شهری یزد انجام گرفته است. به همین منظور، تعداد 142 راننده نوبت کار و 125 نفر غیر نوبت کار به روش تصادفی- سهمیه ای انتخاب گردیدند.  به منظور گردآوری داده های پژوهش، از یک پرسشنامه دو قسمتی فیلیپ ال رایس استفاده شد. سپس داده ها  توسط نرم افزار SPSS مورد تجزیه و تحلیل قرار گرفتند. یافته­ها : آنالیز داده ها نشان داد که میانگین نمره استرس در نوبت کاران، بالاتر از غیر نوبت کاران (03/0P=) می باشد. همچنین میانگین نمره استرس بر حسب نوع بیماری، نشان داد که به غیر از کم خوابی، در بقیه بیماری ها از لحاظ آماری اختلاف معنی داری وجود ندارد. افراد با سابقه بیماری و نوبت کار میزان نمره استرس بالاتری را نشان داده اند و این اختلاف از لحاظ آماری معنی دار  است(013/0P=). نتیجه­گیری : نتایج این تحقیق نشان داد که میانگین نمره استرس بر حسب سابقه بیماری در گروه رانندگان نوبت کار، بالاتر از غیر نوبت کار می باشد. سطح بالای استرس ، ضرورت اجرای یک برنامه جامع مدیریت استرس را در سازمان نشان می دهد تا از میزان بروز استرس و پیامد های آن کاسته شود.

Title: تجزیه و تحلیل سیستم مدیریت ریسک هایHSE و مدل سازی ارتباط آن با حوادث شغلی در صنعت ساخت و ساز

http://tbj.ssu.ac.ir/article-1-2006-fa.html

مقدمه: ریسک حوادث شغلی در صنعت و سایت های ساخت و سازی بسیار بالا و خطرناک می باشد. استقرار یک سیستم مدیریت ریسک کارا و اثربخش ،گامی مهم در راستای پیشگیری از بروز حوادث ساخت و ساز می باشد. هدف از اجرای این مطالعه تجزیه و تحلیل سیستم مدیریت ریسک HSE موجود در سایت های ساخت و سازی بزرگ کشور و بررسی ارتباط آن با حوادث شغلی بود. روش بررسی: ­این مطالعه یک بررسی مقطعی از نوع توصیفی-تحلیلی بوده که به تجزیه و تحلیل سیستم مدیریت ریسک و ارتباط آن با حوادث اتفاق افتاده طی سال های 92-88 در سایت های بزرگ ساخت و ساز کشور پرداخته است. داده های مطالعه شامل اطلاعات مربوط به همه سیستم های مدیریت ریسک HSEو حوادث آسیب زای انسانی بود که طی 5 سال در سایت های مختلف ساخت و ساز در کشور رخ داده بود. تجزیه و تحلیل داده های مطالعه با استفاده از نرم افزار آماری SPSSنسخه 16 انجام گرفت. سطح معنی داری در این مطالعه 05/0=pدر نظر گرفته شد. یافته ها: میانگین شاخص شدت حادثه (ASR) 104×05/1±103×51/1 بود. نتایج مطالعه نشان داد که 4/18% از سایت های مورد مطالعه سیستم مدیریت ریسک، استقرار یافته و سهم اجزا و شاخص های آن شامل شناسایی خطرات، ارزیابی دوره ای ریسک، اجرای روش های کنترلی به ترتیب 4/16%، 6/16% و 2/10%، برآورد گردید. بعلاوه، همه شاخص های مدیریت ریسک با شاخص ASR دارای ارتباط معنی دار بودند (05/0>p). نتیجه گیری: تحلیل رگرسیونی شاخص شدت حادثه (ASR) بر مبنای شاخص های مدیریت ریسک،نشان داد که پارامترهای دموگرافیک و شغلی مانند سابقه کار و نوع شغل و همچنین شاخص های مدیریت ریسک مانند استقرار سیستم مدیریت ریسک، سیستم HAZID، ارزیابی ریسک دوره ای، آموزشHSEو Housekeepingبا شدت پیامد ناشی از حادثه در صنعت ساخت و ساز دارای ارتباط معنی دار می باشند.

Title: مقایسه کارایی بسترهای سرباره‌ای، زئولیتی و معمولی در صافی‌های شنی کند جهت حذف سرب و کادمیوم از منابع آب

http://tbj.ssu.ac.ir/article-1-2008-fa.html

مقدمه: فلزات سنگین به علت مخاطرات بهداشتی و سمیتی که در غلظت‌های کم برای انسان و محیط دارند بسیار مورد توجه هستند. یکی از روش‌های ساده و مقرون به‌صرفه جهت حذف این آلاینده‌ها صافی شنی کند می‌باشد. نوع بستر نقش موثری در حذف آلودگی وارده به این سیستم‌ها را دارد. لذا هدف از این طرح تحقیقاتی بررسی اثر بسترهای مختلفی چون ماسه معمولی، زئولیتی و سرباره‌ای در حذف سرب و کادمیوم می‌باشد. روش بررسی: در این تحقیق از سه فیلتر با بستر ماسه‌ای معمول، سرباره و زئولیتی در مقیاس پایلوت جهت بررسی حذف سرب و کادمیوم در سه غلظت 1/0، 1 و ppm 10 استفاده شده است. فیلترها از جنس پلکسی گلاس و دارای قطر 8 و ارتفاع 120 سانتی متر بوده‌اند که به صورت جریان پیوسته بهره برداری شده‌اند. یافته‌ها: راندمان حذف کدورت برای سه بستر ماسه معمولی، سرباره و زئولیتی با کدورت اولیه NTU 13 به ترتیب 46، 77 و 89% بوده است. راندمان حذف سرب نیز در حالت عدم وجود کدورت و غلظت 1/0 پی پی‌ام سرب به ترتیب 3/70%، 79% و 8/59% بوده و برای غلظت ppm 1 به ترتیب 8/51%، 7/52% و 6/52% بوده و برای غلظت ppm 10 سرب به ترتیب 4/53%، 8/57% و 8/59% بوده است. راندمان حذف کادمیوم نیز در غلظت ppm 1/0 کادمیوم به ترتیب 4/23%، 5/37% و 4/59% بوده وبرای غلظت ppm 1 به ترتیب 9/37%، 45% و 3/41% و در غلظت ppm 10 به ترتیب 3/68%، 6/68% و 67% بوده است. نتیجه­گیری: بسترهای سرباره و زئولیتی راندمان بیشتری در حذف سرب و کادمیوم نسبت به بسترهای ماسه‌ای معمولی دارند لذا جهت حذف فلزات از منابع آبی می‌توان این نوع بسترها را به جای ماسه معمول استفاده نمود.

Title: ارزیابی میزان خستگی شغلی در کارگران صنعت فولاد یزد و ارتباط آن با برخی متغیرهای دموگرافیکی در سال 1393

http://tbj.ssu.ac.ir/article-1-2010-fa.html

مقدمه: خستگی پدیده ای مختص موجودات زنده است، که قطعا همه انسان ها در زندگی معمول خود آن را تجربه کرده و به عنوان یک موضوع با اهمیت محسوب و ممکن است در عملکرد شغلی مورد انتظار کارگران از جمله ضعف در قضاوت و تصمیم گیری، فراموش نمودن جزئیات، بی تفاوتی به موارد ضروری و افت عملکرد آنها اختلال ایجاد کند.  این مطالعه با هدف بررسی میزان شیوع خستگی و تعیین ارتباط آن با برخی متغیرهای دموگرافیکی انجام شد. روش­بررسی: مطالعه حاضر از نوع مقطعی - توصیفی بوده که در سال 1393 در کارخانه فولاد یزد با حجم نمونه 388 نفر  که به صورت تصادفی خوشه ای انتخاب شدند انجام شده است. جمعآوری داده ها به صورت حضوری و با استفاده از پرسشنامه مقیاس بازیابی خستگی شغلی ( OFER ) و همچنین پرسشنامه اطلاعات دموگرافیکی انجام شد. داده ها با استفاده از نرم افزار19 SPSS ،تجزیه و تحلیل شدند. یافته­ها: نتایج این مطالعه نشان داد که بیشتر افراد مورد مطالعه، شدت متوسطی از خستگی مزمن و در مورد خستگی حاد، بیشتر افراد شدت زیادی را تجربه کرده اند. شیوع خستگی شدید مزمن و حاد به ترتیب 49/30 درصد و 4/55 درصد مشاهده شد.  ارتباط معناداری بین خستگی با سابقه شغلی و سطح تحصیلات به دست آمد( 019/0(P-value=. نتیجه­گیری: نتایج حاصل از این مطالعه حاکی از شیوع بالای خستگی در جمعیت مورد مطالعه می باشد.

Title: پیش بینی کننده های کنترل فشار خون بالا بر اساس مدل اعتقاد بهداشتی در بیماران مبتلا به پرفشاری خون شهر کرمان در سال 1394

http://tbj.ssu.ac.ir/article-1-1995-fa.html

مقدمه: شیوع بالای فشار خون در سراسر جهان و ایجاد عوارض جدی و خطرناک بر ارگان های بدن، این بیماری را به یک مشکل عمده بهداشتی در کلیه جوامع تبدیل کرده است که مدیریت این بیماری بسیار مهم به نظر می رسد. مطالعه حاضر با هدف تعیین عوامل پیش بینی کننده فشار خون بالا بر اساس مدل اعتقاد بهداشتی در بیماران مبتلا به پرفشاری خون انجام یافت. روش بررسی: این مطالعه توصیفی-تحلیلی از نوع مقطعی بر روی 135 نفر از بیماران فشار خونی بالای 30 سال در شهر کرمان انجام شد. نمونه‌گیری به روش چند‌مرحله‌ای انجام گرفت. بیماران به صورت نمونه‌گیری تصادفی ساده وارد مطالعه شدند. پرسش‌نامه‌ها به صورت خودگزارش‌دهی و مصاحبه تکمیل شد. ابزار جمع‌آوری داده‌ها پرسشنامه عوامل دموگرافیک، سؤالات آگاهی و مدل اعتقاد بهداشتی بود. برای تجزیه و تحلیل داده ها از نرم افزار SPSS نسخه 15 و برای آنالیز نتایج شاخص های آماری و رگرسیون خطی استفاده شد. یافته‌ها: میانگین سن افراد 47/10 ±45/46 سال بود. از بین متغیرهای دموگرافیک ،سن وضعیت تاهل ارتباط معنا دار مستقیمی با کنترل پرفشاری خون در افراد داشتند. آزمون آماری رگرسیون بین سازه‌های خودکارآمدی، منافع درک شده و راهنما برای عمل با میزان فشار خون رابطه معنی‌داری نشان داد (001/0 (P<.  نتیجه‌گیری: با توجه به نتایج میانگین امتیاز کسب شده از سازه های مورد بررسی مدل اعتقاد بهداشتی در حد متوسط بود. پیشنهاد می شود یک برنامه ریزی دقیق آموزشی برای بیماران فشار خونی بر اساس مدل اعتقاد بهداشتی بر روی سازه های خودکارامدی، منافع درک شده و راهنما برای عمل که پیش گویی کننده های قوی فشار خون بودند، انجام گیرد تا در دراز مدت بتوانیم از عوارض این بیماری جلوگیری کنیم.

Title: اثرات مصرف مکمل گیاه چای ترش بر فشار خون و آلبومین اوری در بیماران مبتلا به نفروپاتی دیابتی

http://tbj.ssu.ac.ir/article-1-1997-fa.html

مقدمه: نفروپاتی دیابتی شایعترین علت نارسایی کلیوی است.فشار خون بالا به ویژه افزایش فشارخون گلومرولی یکی از عوامل زمینه ساز ابتلا به نفروپاتی در بیماران دیابتی است. مطالعه حاضر به منظور بررسی اثرات تجویز مکمل گیاه چای ترش بر فشار خون و آلبومین ادری در بیماران مبتلا به نفروپاتی دیابتی انجام شد. روش­بررسی: این مطالعه یک کارآزمایی بالینی تصادفی دو سوکور بود که بر روی 41  بیمار مبتلا به نفروپاتی دیابتی (15 مرد 26 زن) انجام شد. بیماران به صورت تصادفی به دو گروه دریافت کننده مکمل گیاه چای ترش (روزانه دو قرص 425 میلیگرمی) و دارونما تقسیم شدند. مدت مطالعه 8 هفته بود. در شروع مطالعه و پایان هفته هشتم یک نمونه ادرار تصادفی وcc5 خون بعد از 12 تا 14 ساعت ناشتایی گرفته شد وفشار خون سیستولیک و دیاستولیک و غلظت آلبومین ادرار اندازه گرفته شد. یافته ها: فشار خون سیستولیک در گروه دریافت کننده مکمل گیاه چای ترش در پایان هفته هشتم نسبت به زمان شروع مطالعه از 135 به 5/119 کاهش پیدا کرد که این کاهش از نظر آماری معنی دار بود (05/0(p. در پایان هفته هشتم در گروه دریافت کننده مکمل گیاه چای ترش غلظت آلبومین ادراری نسبت به شروع مطالعه mg20کاهش پیدا کرد که این کاهش از نظر آماری معنی دار بود ( 05/0p (. تغییرات مشاهده شده در متغیرهای ذکر شده در گروه دارونما معنی دار نبود. نتیجه گیری:به طور کلی مطالعه حاضر نشان می دهد که تجویز مکمل گیاه چای ترش سبب کاهش معنی دار فشار خون سیستولیک و آلبومین اوری می شود. بنابراین تجویز  مکمل گیاه چای ترش ممکن است در کند کردن پیشرفت نفروپاتی دیابتی نقش موثری داشته باشد.

Title: بررسی عوامل موثربر فشار خون بالا در جمعیت روستایی بالای 30 سال-شهرستان اسلام آباد غرب-1392

http://tbj.ssu.ac.ir/article-1-1998-fa.html

مقدمه: یکی از مهمترین بیماریهای غیر واگیر در تمام گروههای سنی ، پر فشاری خون است و اغلب بدون علامت، سریعأ قابل تشخیص ودرمان پذیراست واگر درمان نشود ممکن است منجر به عوارض کشنده شود. این مطالعه به منظور "بررسی علل فشار خون بالا در جمعیت روستایی بالای 30سال شهرستان اسلام آباد غرب "  انجام گرفت. روش بررسی :نوع مطالعه دراین پژوهش توصیفی –تحلیلی  می باشد که به منظور تشخیص واهمیت ریسک فاکتورهای فشار خون بالا انجام گرفت جامعه مورد مطالعه 320نفر از بیماران فشارخون بالادر جمعیت روستایی بالای 30سال شهرستان اسلام آباد غرب میباشند.که بصورت تصادفی ساده از بین بیماران فشارخونی انتخاب شده اند .ابزار گرد آوری داده ها پرسشنامه بود که روایی آن توسط کارشنا سان تأ یید وجهت پایایی 5% ازافراد مورد بررسی  انتخاب شدند تا به سوالات پرسشنامه پاسخ دهند، وبا مشاهده اشکالات موجود در پرسشنامه تغییرات اصلاحی صورت پذیرفت.داده ها با استفاده از نرم افزار SPSS وآزمونهای paired t-test,chi2 تجزیه وتحلیل شد. یافته ها: از مجموع 320نفر ازبیماران مورد بررسی 109 نفر مرد و211نفر زن بودند و فشارخون بالادرزنان بالای 50سال ، متأهل وخانه داربیشتر از مردان بود .81%مردان و82% زنان دارای فشارخون بیسواد هستند وحدود 78% ازجمعیت مورد مطالعه نمک زیادی مصرف می کنند میزان مصرف مواد غذایی مثل ماهی بسیار کم ومیزان مصرف میوه وسبزیجات خوب بوده است. ( 96%)، 74%از افراد مورد مطالعه دارای سابقه خانوادگی  فشارخون بالا و12% مبتلا به دیابت بودند که آمار زنان دراین مطالعه 3برابر مردان است.. درحدود 15%مردان و23% زنان سابقه چربی خون بالا دارند و8%مردان و13% زنان دارای بیماری کلیوی هستند نتیجه گیری:  شیوع فشار خون در جمعیت روستایی با توجه به عدم مصرف مرغ وماهی واستفاده از گوشت قرمز ومصرف نمک زیاد در بین  جمعیت مورد مطالعه بالامی باشدودر زنان شیوع بالاتر است  برای کنترل فشار خون در این جمعیت استفاده از آموزش های  چهره به چهره (با توجه به سطح سواد پایین آنها)و ترغیب مردم به استفاده کمتر از نمک در رژیم غذایی خود و همچنین جایگزین کردن گوشت ماهی و مرغ به جای گوشت قرمز،و بررسی و معاینه افراد در دوره هایی بیشتر از دوره های روتین که تیم سلامت مرکز انجام میدهند.

Title: بررسی تاثیر آموزش فعالیت بدنی مبتنی بر الگوی فرانظری، بر کنترل فشارخون بیماران مبتلا به فشار خون بالا ن

http://tbj.ssu.ac.ir/article-1-1999-fa.html

مقدمه: شیوع بالای فشارخون در سراسر جهان و ایجاد عوارض جدی، مشکل عمده کلیه جوامع، است. برای کاهش عوارض، کنترل فشارخون از اهمیت خاصی برخوردار است. فعالیت بدنی منظم، یکی از موثرترین استراتژی ها برای کاهش خطر  بیماری های غیر واگیر از جمله بیماریهای قلبی عروقی می باشد. پژوهش حاضر با هدف تعیین تاثیر آموزش فعالیت بدنی مبتنی بر الگوی فرانظری، برکنترل فشارخون انجام شد. روش­بررسی: در این مطالعه نیمه تجربی 60 فرد مبتلا به فشارخون بالا با استفاده از روش نمونه گیری در دسترس انتخاب و به طور تصادفی به دو گروه مداخله و کنترل تقسیم گردیدند. ابزار گردآوری داده ها، پرسشنامه ای چند وجهی، مشتمل بر مشخصات دموگرافیک، سوالات مراحل تغییر و عوامل تاثیرگذار بر الگوی فرانظری و پرسشنامه سنجش فعالیت بدنی فرم کوتاه بود. پرسشنامه ها برای دو گروه تکمیل و سپس مداخله آموزشی در گروه مداخله انجام شد. پس از دو ماه مجدداً پرسشنامه ها برای دو گروه تکمیل گردید. داده ها در نرم افزار SPSS تجزیه و تحلیل و آزمون های تی و کای دو با توجه به توزیع داده ها استفاده شد. یافته­ها: آزمونهای آماری اختلاف معناداری در دو گروه قبل از آموزش از نظر مراحل تغییر، موازنه تصمیم گیری، فعالیت بدنی، فشارخون و فرایندهای تغییر،نداشت اما پس از آموزش، افزایش معناداری در کلیه متغیرهای فوق در گروه مداخله نسبت به گروه کنترل به وجود آمده بود. (P<./005). نتیجه گیری: آموزش و مداخله بر پایه الگوی فرانظری باعث ارتقاء رفتار فعالیت بدنی در بیماران و کاهش فشارخون بالامی شود.

Title: بررسی میزان فعالیت فیزیکی در بیماران مبتلا به پرفشاری خون در شهر یزد: یک مطالعه ی مورد شاهدی

http://tbj.ssu.ac.ir/article-1-2005-fa.html

مقدمه: فعالیت بدنی کافی و منظم از عوامل اصلی حفظ و ارتقا سلامت در سراسر دوران زندگی است. نتایج تحقیقات جهانی نشان داده است ورزش منظم و در حد اعتدال سبب افزایش طول عمر و کاهش ابتلا و مرگ ناشی از بیماری ها­ی مزمن می­شود. لذا مطالعه ی حاضر همگام با سایر بررسی ها­ی انجام شده در جهان و ایران با هدف بررسی میزان فعالیت فیزیکی در افراد مبتلا به پرفشاری خون و مقایسه­ی آن با افراد سالم انجام شد. روش بررسی: مطالعه­ی حاضر یک مطالعه­ی مورد- شاهدی است که در سال 1394 بر روی 160 نفر (80 نفر گروه شاهد، 80 نفر گروه مورد) انجام شد. افرادی که دارای فشار خون سیستولیکmmHg 140≤ و یا فشار خون دیاستولیک mmHg90≤ بودند در گروه مورد و افراد با فشار خون نرمال در گروه شاهد قرار گرفتند. روش نمونه گیری به صورت تصادفی آسان بود. داده ها با استفاده از پرسشنامه جمع آوری گردید. قسمت اول پرسشنامه مربوط به اطلاعات زمینه ای و اجتماعی – اقتصادی و قسمت دوم مربوط به سنجش فعالیت فیزیکی با استفاده از پرسشنامه استاندارد IPAQ بود. داده ها پس از جمع آوری با آزمون های Chi-square،  Mann- Whitneyو رگرسیون لجستیک در نرم افزار SPSS/16 تجزیه و تحلیل شد. یافته­ها : بررسی میزان فعالیت فیزیکی در گروه مورد و شاهد نشان داد 8/13 درصد از گروه مورد و 5/27 درصد از گروه شاهد دارای فعالیت فیزیکی بودند و این اختلاف از نظر آماری معنادار بود. بر اساس نتایج بدست آمده از مطالعه شانس ابتلا به پرفشاری خون در گروه بدون فعالیت فیزیکی 37/2 برابر بیشتر از گروهی بود که در طول هفته تحرک بدنی داشتند. نتایج رگرسیون لجستیک نشان داد در بین متغیرهای مورد بررسی، فعالیت فیزیکی، سطح تحصیلات و درآمد عوامل تاثیر گذار بر پرفشاری خون بودند. نتایج نشان داد فعالیت فیزیکی با سطح متوسط و شدید در ابتلا به پرفشاری خون اثر محافظتی دارند. همچنین افراد با سطح تحصیلات پایین تر و درآمد کمتر شانس ابتلا به پرفشاری خون بیشتری را داشتند. نتیجه­گیری: یافته ها­ی پژوهش نشان داد که ورزش و فعالیت فیزیکی شانس ابتلا به پرفشاری خون را کاهش می دهد. این یافته بر ضرورت انجام اقدامات مداخله ای جهت بهبود سبک زندگی مردم تاکید دارد.

Title: مطالعات سینتیک و ایزوترم جذب متیلن بلو از محلولهای آبی توسط خاکستر فرار فعال شده با اسید

http://tbj.ssu.ac.ir/article-1-2002-fa.html

مقدمه: فاضلابهای رنگی به دلیل ایجاد مشکلات زیست محیطی گوناگون باید قبل از تخلیه به محیط زیست، تصفیه شوند. رنگها تنوع بسیاری دارند و از بین روشهای متفاوت بکار رفته جهت تصفیه فاضلابهای رنگی، فرایند جذب با جاذب ارزان قیمت از پرکاربردترین روشها می باشد. به منظور تعیین ایزوترم و سینتیک جذب ،از جذب رنگ کاتیونی متیلن بلو  بعنوان رنگ شاخص با استفاده از خاکستر فرار فعال شده نیروگاه برق حرارتی شهرستان زرند، این تحقیق انجام گردید. روش­بررسی: در این تحقیق تجربی خاکستر فرار خام در زمان 5 ساعت  غلظت اسید 8/1 مولار، دمای C˚2±29 و نسبت اسید به خاکستر فرار برابر با 7 توسط اسید سولفوریک فعال گردید. پس از تعیین مقادیر بهینه پارامترهای موثر در فرایند جذب (pH، زمان تماس، غلظت جاذب) بمنظور تفهیم بهتر فرایند جذب ایزوترم های فروندلیچ و لانگمیر نوع1، 2، 3 و 4 و سینتیک درجه اول و دوم نوع 1، 2، 3 و 4 و  فاکتور تفکیک بررسی شد. یافته­ها:خاکستر فرار فعال شده در شرایط بهینه )9=pH، زمان 30 دقیقه، مقدار جاذب3 گرم بر لیتر)مقدار 8/99% از رنگ متیلن بلو در غلظت 50 میلی گرم بر لیتر را حذف نمود. فرایند از سینتیک درجه دوم نوع 1 با 1=R و ایزوترم لانگمیر نوع 1 با 997/0=R تبعیت می کند. نتیجه­گیری: با توجه به اینکه مقدار فاکتور تفکیک و شدت جذب سطحی در محدوده بین 0 و 1 قرار دارد بنابراین  فرایند جذب، مطلوب می­باشد. لذا خاکستر فرار فعال شده بعنوان یک جاذب قوی در جذب رنگ متیلن بلو عمل میکند.

Title: ارزیابی کمی و کیفی فاضلاب ترمینالهای ضبط پسته (مطالعه موردی شهرکرمان)

http://tbj.ssu.ac.ir/article-1-2026-fa.html

مقدمه: فاضلاب­های ناشی از فرآوری­های فصلی از جمله صنعت فرآوری پسته، یکی از عوامل مهم آلودگی محیط محسوب می­شوند. مشخصه کیفی این فاضلاب­ها، غلظت زیاد مواد آلاینده در آنهاست که به علت مصرف  بالای آب و منابع انرژی و نیز میزان بالای تولید پساب پتانسیل خوبی را برای اجرای پروژه­های کاهش آثار زیست محیطی و بهینه­سازی مصرف منابع انرژی دارا می­باشند. هدف از تحقیق تعیین کمیت و کیفیت فاضلاب ترمینال­های ضبط پسته شهر کرمان می­باشد. روش بررسی: تحقیق توصیفی-مقطعی است که از اواسط شهریورماه لغایت اوایل آبان­ماه (فصل برداشت پسته) در هریک از سال­های91-92-93 در شهر کرمان به اجرا درآمده است. ابتدا با بازدیدهای میدانی، مشخصات و مکان جغرافیایی20 ترمینال ضبط پسته بوسیله دستگاه جی پی اس بصورت سرشماری تعیین گردید. از بین ترمینالهای مذکور 8 ترمینال اطراف خطوط جمع آوری فاضلاب شهر انتخاب شد. سپس نمونه برداری بصورت مرکب با مجموع دفعات 72 نمونه در هرسال در دوره فرآوری پسته از خروجی آشغالگیر فیلتری انجام شد. در هریک از نمونه ها میزان BOD5،COD ،TSS ،pH ، Total phenolic  تعیین گردید. نمونه برداری و آزمایشات منطبق بر دستورالعمل کتاب آزمایشات آب و فاضلاب چاپ بیستم صورت گرفت.غلظت مواد فنولیک به روش فولین سیکالتیو اندازه گیری شد. تجزیه و تحلیل داده ها با استفاده از نرم افزار SPSS انجام شد. یافته­ها: میانگین کل فاضلاب تولیدی ترمینال­های ضبط پسته مورد مطالعه،9/85 مترمکعب در روز برآورد شد. میانگین میزان  پارامترهای  BOD5،COD،TSS،Total Phenolic،pH در آنهابه ترتیب 6106، 21570،682، 4154 mg/L و 5/5 می باشد. نتیجه­گیری: نتایج حاصل از تعیین کیفیت فاضلاب خام ترمینال­های ضبط پسته نشان داد که این فاضلاب ­دارایBOD و COD بالا می باشد.علت آن وجود آلاینده های مقدم«ترکیبات فنولیک» درآنهااست که دارای پتانسیل بالای آلودگی و  سمیت زیاد برای تخلیه به هر منبع پذیرنده می باشد.

Title: بررسی خصوصیات شیمیایی و درجه رسیدگی ورمی کمپوست بدست آمده از پسماندهای فرایند تولید قارچ دکمه ای

http://tbj.ssu.ac.ir/article-1-2009-fa.html

مقدمه : در ایران، توسعه صنعت پرورش قارچ خوراکی مورد توجه قرار گرفته و میزان تولید آن به نود هزارتن در سال رسیده است. با تولید هرکیلوگرم قارچ خوراکی دکمه ای، حدود پنج کیلوگرم پسماند بر جای می ماند که با استفاده از تکنولوژی ورمی کمپوست می توان ضمن تصفیه این پسماندها آن را به کود آلی با ارزشی تبدیل نموده و میزان استفاده کودهای شیمیایی را کاهش داده و در جهت توسعه پایدار گام برداشت.هدف این مطالعه تبدیل پسماندهای فرایند تولید قارچ دکمه ای به کود ورمی و بررسی خصوصیات شیمیایی و کیفیت این کود می باشد. روش بررسی: طی فرایند تولید قارچ دکمه ای( آگاریکوس )، پسماندهایی همچون زائدات قارچ و خاک پیت و کمپوست مصرف شده بر جای می ماند که حجم بالائی از مواد زائد را تشکیل داده که به آن پسماند کمپوست قارچ مصرف شده می­گویند. در این تحقیق ضمن انجام فرایند ورمی کمپوست روی پسماندهای کمپوست قارچ مصرف شده یا SMC در شرایط بهینه با تیمارهای چیپس چوب،ضایعات قارچ،برگ وتفاله هویج، شاخص های کمی و کیفی و تغییرات آن طی فرایند شش هفته ای مورد بررسی قرار گرفت و نتایج بدست آمده با استاندارد ملی ورمی کمپوست (شماره 13724) مقایسه گردیده و درجه کیفیت محصول نهایی تعیین شده   و با استفاده از نرم افزار SPSS ،آزمون  همبستگی پیرسون در سطح معنی داری 05/0 جهت بررسی ارتباط روند معدنی شدن مواد و تغییرات سایر پارامترها و نیز از آزمون رگرسیون خطی جهت یافتن رابطه بین یک متغیر و سایر متغیرها ، بهره گرفته شد. یافته ها: نتایج بدست  آمده نشان داد که کل کربن آلی (TOC) ، پتاسیم و نیتروژن کل،  در همه تیمارها با گذشت زمان افزایش یافته اما میزان pH وEC ،طی فرایند ورمی کمپوست کاهش یافته است.همچنین افزایش معنی داری، در نسبت C/N در همه تیمارها در انتهای فرایند شش هفته ای مشاهده شد و تمامی کودهای نهایی در ردیف کودهای درجه یک قرار گرفتند. نتیجه گیری: نتایج این پژوهش نشان داد که ورمی کمپوست منجر به بهبود شاخص های کود آلی دربسترهای تحت فرایند ورمی کمپوست گردیده و یکی از روشهای مناسب مدیریت برای پسماندهای کارگاههای تولید قارچ دکمه ای می باشد که راهی در جهت نیل به کشاورزی پایدار محسوب می شود

Title: پراکنش و اثرات زیست محیطی آلودگی صوتی فرودگاه بین المللی شهید صدوقی یزد با استفاده از GIS

http://tbj.ssu.ac.ir/article-1-2012-fa.html

مقدمه: آزاردهندگی حاصل از سروصدای هواپیماها در یک فرودگاه، رابطه نزدیکی با تناوب نشست و برخاست هواپیماها و توزین برنامه ریزی عملیات آنها در طول شبانه­ روز دارد. در حال حاضر هواپیماهایی که در فرودگاه یزد نشت و برخاست می کنند از نوع هواپیماهای متوسط با موتورهای جت یا اصطلاحاً توربوفن بوده که صدای حاصله از آنها بسیار زیاد خواهد بود. روش بررسی: این تحقیق توصیفی تحلیلی، بصورت مقطعی بوده و اندازه­گیری میزان سروصدای ناشی از تردد هواپیما در 7 ایستگاه سنجش صوت توسط دستگاه سنجش صوت سیار TesTo-  815 و همچنین تکمیل 100 پرسشنامه جهت بررسی آثار ناشی از آلودگی صوتی  در فرودگاه بین المللی یزد و مناطق حساس صوتی اطراف آن در سال 1393 انجام شده است. یافته­ها: بیشترین میزان آلودگی صوتی ناشی از تردد هواپیما ،در خود محوطه فرودگاه با میانگین تراز صوتی 8/84 دسی­بل و در مناطق مجاور فرودگاه در ایستگاه شماره 5 منطقه­ای واقع در جنوب فرودگاه، با میانگین تراز صوتی 5/75 دسی­بل بدست آمد. نتایج بدست آمده از آنالیز پرسشنامه­ها نشان داد که 75 درصد مردم ساکن مناطق مسکونی اطراف فرودگاه بوسیله سروصدای هواپیما مورد آزار و اذیت قرار می گیرند. نتایج بیانگر آن است که، اختلال در خواب، حالت عصبی، استرس و بیماری روحی و تداخل در گفتار از عواملی هستند که صرف نظر از اولویت بندی متفاوتشان در منطقه مورد بررسی، از بیشترین میزان تأثیرپذیری از نویز هواپیماها برخوردار هستند. نتیجه­گیری: سروصدای هواپیما قویترین فاکتور زیست محیطی منفی می باشد که کارکنان و ساکنین اطراف فرودگاه بین المللی یزد را تحت تأثیر قرار داده و میتواند برای سلامت آنها خطرناک باشد. این سروصدا برای سلامتی کارمندان بویژه آنان که بواسطه نوع وظایفشان، روزانه در معرض سروصدای شدید هواپیماها قرار دارند، بسیار نامطلوب است. از این رو ضرورتاً ضوابط پیشگیرانه شدیدی مانند استفاده اجباری از وسایل حفاظتی آکوستیک و ضد صدا یا کوتاه کردن زمان خدمت کارکنان، برای این گونه افراد مورد نیاز است

Title: حذف استامینوفن از محلول‌های آبی توسط فتوکاتالیست TiO2/زئولیت X

http://tbj.ssu.ac.ir/article-1-2007-fa.html

مقدمه: پسماندهای دارویی در گروه آلاینده­های نوظهور قرار دارند. این مواد با روش­های متعارف تصفیه ی آب و فاضلاب، بهطورکامل حذف نمی­شوند. داروها و متابولیت­های آن ها­، به­علّت ایجاد خطرات احتمالی و سمیّت برای انسان و محیط، بایستی از منابع آبی حذف گردند. در این تحقیق، حذف فتوکاتالیستی استامینوفن از محلول­های آبی، توسط زئولیت X اصلاح شده با TiO2 (TiO2/زئولیت X)، مورد بررسی قرار گرفت. روش­بررسی: روش این تحقیق، تجربی بوده و در مقیاس آزمایشگاهی انجام شد. جهت حذف استامینوفن از محلول­های آبی، از فتوکاتالیست TiO2/زئولیت X، تحت تابش UV-C (فرآیند UV/TiO2-X) استفاده گردید. غلظت استامینوفن محلول­ها با روش طیف­سنجی فرابنفش-مرئی در  طول موج 243 نانومتراندازه­گیری شد. شرایط بهینه از نظر pH محلول، دوز فتوکاتالیست، غلظت استامینوفن و زمان واکنش تعیین و سینتیک تجزیه، مورد بررسی قرار گرفت. یافته­ها: نتایج نشان داد که بیشترین حذف استامینوفن (45/95%) مربوط به غلظت اوّلیۀ 1­ میلی گرم بر لیتر در pH خنثی و 500 میلی گرم بر لیتر دوز کاتالیست پس از گذشت زمان ­75 دقیقه از شروع واکنش بوده است. با کاهش غلظت استامینوفن میزان حذف آن، افزایش یافت. سرعت واکنش تجزیه، از مدل سینتیک درجه اوّل کاذب با ثابت سرعت min-1 01446/0 پیروی نمود. نتیجه­گیری: روش فتوکاتالیستی UV/TiO2-X در حذف استامینوفن از محلول­های آبی، دارای کارایی بسیار بالایی بوده و قابل توصیه است.

Title: بررسی کارایی نانولوله‌های کربنی چند دیواره در حذف رنگ اسید بلاک 1 از محلول های آبی

http://tbj.ssu.ac.ir/article-1-2019-fa.html

مقدمه: پساب رنگی برخی صنایع شامل تعدادی ترکیبات سرطان زا،  سمی و یا جهش زا می باشد ، بنابراین آلاینده های رنگی فاضلاب این نوع صنایع، باید قبل از تخلیه به محیط زیست با روش مناسب تصفیه گردند. هدف از انجام این پژوهش بررسی کارآیی نانو تیوب کربن چند دیواره ،در حذف رنگ اسید بلاک 1 از  فاضلاب رنگی  بوده است. روش­بررسی : این پژوهش یک مطالعه آزمایشگاهی بودکه در سیستم ناپیوسته انجام گردید واز نانو تیوب کربن چند دیواره، به عنوان جاذب برای حذف رنگ  استفاده شد. دراین مطالعه عوامل مختلف تاثیر گذار روی میزان حذف رنگ مانند دوز جاذب، غلظت اولیه رنگ، زمان تماس و pH  بررسی شد. یافته­ها :نتایج نشان داد  مناسب ترین pH  برای حذف رنگ،3 می باشد و زمان تعادل برای جذب رنگ اسید  بلاک 1روی جاذب  برابر 60 دقیقه بود. با افزایش غلظت رنگ، سرعت حذف رنگ کاهش یافت. افزایش مقدار جاذب باعث افزایش راندمان حذف رنگ شد بطوریکه در دوز 600 میلی گرم بر لیتر، راندمان حذف رنگ با غلظت 30  و50 میلی گرم بر لیتر به ترتیب برابر86/98 و 62/94 درصد به دست آمد.همچنین حذف رنگ اسید بلاک1 ،از مدل ایزوترمی لانگمویر و سینتیک درجه  دوم تبعیت کرد. نتیجه­گیری: نتایج مطالعات جذب ، نشان داد که با افزایش زمان تماس و دوز جاذب و کاهش, pH  راندمان حذف رنگ افزایش یافته و نانو لوله‌های کربنی چند جداره  به عنوان یک جاذب موثر برای حذف رنگ های آزو از فاضلاب می توانند استفاده شوند.

Title: کاربرد فرآیند فتوکاتالیستی Ag-ZnO/UV-Cدر تجزیه سم 2, 4-دی کلروفنوکسی استیک اسید در محلول های آبی

http://tbj.ssu.ac.ir/article-1-2021-fa.html

مقدمه: انواع گوناگونی از سموم به منظور مبارزه با علف های هرز مورد استفاده قرار میگیرند. ورود بقایای این آلاینده های مقاوم به منابع تأمین آب شرب میتواند اثرات مخربی بر سلامت انسان و محیط زیست داشته باشد. در سال های اخیر استفاده از روشهای اکسیداسیون پیشرفته برای حذف سموم از محیط زیست به ویژه محیط های آبی، بیشتر مورد توجه قرار گرفته است. روش بررسی: دراین تحقیق، نمونه های مورد نظر با اضافه نمودن غلظت های (mg/L30 ،20 ،10) از سموم مورد مطالعه به آب دیونیزه تهیه گردید. نمونه ها در مراحل جداگانه در سیستم ناپیوسته با مقدار کاتالیزور (g/L 4 و3 ،2 ،1)، pH (11 ،9 ،7 ،5 ،3) و  زمان تماس (240 ،180 ،120 ،60 دقیقه) درمعرض تماس توأم Ag/ZnO و UV-C قرارگرفتند. یافته ها: براساس نتایج با کاهش غلظت سم (2, 4-D) و افزایش زمان واکنش، راندمان تجزیه افزایش مییابد. و بیشترین راندمان تجزیه برای زمان 240 دقیقه و غلظت اولیه سم (2, 4-D) mg/L 10 برابر 96 درصد بوده است. همچنین مطالعه سینتیک تجزیه سم (2, 4-D) توسط فرآیند فتوکاتالیستی Ag/ZnO در حضور نور UV-c با مدل شبه درجه اول مطابقت دارد. نتیجه گیری: بیشترین کارایی تجزیه سم (2, 4-D) توسط فرآیند فتوکاتالیستی Ag/ZnO تحت شرایط pH خنثی و با افزایش زمان واکنش و کاهش غلظت سم (2, 4-D) حاصل میشود.

Title: بررسی کیفیت زندگی مرتبط با سلامت در زنان و دختران مبتلا به سندرم تخمدان پلی کیستیک شهر یزد ، سال 1393

http://tbj.ssu.ac.ir/article-1-2011-fa.html

مقدمه: سندرم تخمدان پلی کیستیک یکی از شایع ترین اختلالات غدد درون ریز زنان در سنین باروری  است.شیوع این بیماری در حدود 15-4 در صد می باشد. بیماری معمولا با اختلالات قاعدگی،هیرسوتیسم،آکنه، چاقی و در طولانی مدت با عوارض متابولیک همراه می باشد و کیفیت زندگی افراد مبتلا را تحت تاثیر قرار     می دهد. هدف از این مطالعه  اولویت بندی مشکلات بیماران مبتلا به سندرم تخمدان پلی کیستیک برای کمک به برنامه ریزی در جهت بهبود کیفیت زندگی افراد مبتلا می باشد. روش بررسی: این مطالعه از نوع توصیفی به روش مقطعی بر روی70  زن و دختر مبتلا به سندرم تخمدان پلی کیستیک مراجعه کننده به کلینیک تخصصی بقایی پور شهر یزد ،در سال 1393  انجام شد. ابزارگردآوری داده ها  پرسشنامه کیفیت زندگی مختص زنان مبتلا به سندرم تخمدان پلی کیستیک بود.داده ها با تکمیل پرسشنامه کیفیت زندگی ویژه زنان مبتلا به سندرم تخمدان پلی کیستیک جمع آوری گردید. از آمار توصیفی و استنباطی و سطح معنی داری 05/0، جهت تجزیه و تحلیل داده ها استفاده شد. یافته ها : میانگین نمرات کسب شده به ترتیب از کمتر به بیشترشامل:اختلالات قاعدگی(57.6)،وزن(63.06)،ناباروری(68.77)،عاطفی(68.9) و هیرسوتیسم(6/72) می باشد. میانگین سنی 6.14±29.03و میانگین BMI 6/5 ± 3/28 بود. بیش از 70 درصد دارای BMI بالاتر از 25 بودند. ازمیان اطلاعات دموگرافیک، سن، وضعیت تاهل، سطح تحصیلات، میزان درآمد و وضعیت اشتغال در هیچکدام از حیطه ها تاثیری نداشتند. نمره کیفیت زندگی بر اساس BMI تنها در حیطه وزن (P value:./001)و بر اساس داشتن و نداشتن فرزند تنها در حیطه ناباروری اختلاف معنی دار وجود داشت .(P value: ./000) نتیجه­گیری: با توجه به تاثیر عمیق این بیماری بر جنبه های مختلف  سلامت  افراد  مبتلا، نیاز به انجام مطالعات بیشتر برای بررسی ارتباط بین علائم و درمان های انجام شده بر کیفیت زندگی افراد و برنامه ریزی  برای افزایش کیفیت زندگی افراد  ضروری به نظر می رسد.

Title: کارآیی آموزش بر اساس مدل اعتقاد بهداشتی بر رفتارهای پیشگیری کننده از مواجهه با آفت کش در کشاورزان

http://tbj.ssu.ac.ir/article-1-2014-fa.html

مقدمه:استفاده غیراصولی از آفت کش،می تواند منجر به بروزمشکلات متعددی در کشاورزان  بویژه در کشورهای در حال توسعه از جمله ایران شود. با توجه به ماهیت زیست کشی، تأثیرات منفی آفت کش غیر قابل اجتناب بوده ویکی ازمهمترین روش پیشگیری از عوارض آن، افزایش آگاهی می باشد.این مطالعه با هدف بررسی کارآیی مدل اعتقاد بهداشتی در ارتقاء رفتارهای پیشگیری کننده از مواجهه با آفت کش انجام گردید. روش­بررسی:در یک مطالعه مداخله ای،شهرستان انار به دو منطقه غیرمداخله(دهستان بیاض) و منطقه مداخله (دهستان امین شهر) تقسیم شده، و تعداد 128 نفراز کارگران (54 نفر درگروه مداخله و54نفردرگروه غیر مداخله به روش تصادفی انتخاب شدند.گروه مداخله در دو جلسه آموزش بر مبنای مدل اعتقاد بهداشتی شرکت کردند. اطلاعات از طریق تکمیل پرسشنامه طی مصاحبه مستقیم و در دو نوبت، قبل و دو ماه بعد از آموزش جمع آوری گردید. روایی و پایایی سازه های پرسشنامه با مطالعه مقدماتی به اثبات رسید.سپس داده ها با استفاده از نرم افزار 18SPSS مورد تجزیه و تحلیل قرار گرفت. یافته ها: دوگروه از نظر مشخصات فردی تفاوت معنی داری نداشتند. بعد از آموزش، درگروه مداخله افزایش معنی داری در میانگین نمرات آگاهی (002/0=P) و میانگین نمرات حساسیت، شدت، منافع، خودکارآمدی، راهنما برای عمل درک شده(000/0=P)مشاهده گردید. نمره میانگین موانع درک شده (004/0=P)کاهش ومیانگین نمره رفتارهای پیشگیری کننده افزایش معنی دار یافت(000/0=P). نتیجه گیری:با توجه به نتایج مطالعه و کارآیی مدل اعتقاد بهداشتی بررفتارهای پیشگیری کننده ازمواجهه باآفت کش درکشاورزان،تدوین و اجرای برنامه استراتژی جامع جهت ارتقاء رفتارهای ایمن ضروری است.

Title: بررسی آگاهی، نگرش، عملکرد و وضعیت بهداشت دهان و دندان دانش آموزان شهر یزد

http://tbj.ssu.ac.ir/article-1-2016-fa.html

مقدمه: سلامت دهان و دندان یکی از مهمترین عوامل مؤثر در سلامت عمومی می باشد. با توجه به شیوع بالای پوسیدگی دندان و درمان پر هزینه آن و نقش مستقیم فرد در پیشگیری از پوسیدگی دندان لازم است دانش آموزان، آگاهی کافی و نگرش مناسب برای رعایت بهداشت دهان و دندان به دست آورند. باتوجه به کمبود مطالعات جامع در این زمینه، این مطالعه با هدف بررسی آگاهی، نگرش، عملکرد و وضعیت بهداشت دهان و دندان دانش آموزان دبیرستانی شهر یزد انجام شد. روش بررسی: این مطالعه مقطعی  بر روی 300 دانش آموز پسر دبیرستانی شهر یزد که به روش نمونه گیری خوشه ای انتخاب شدند، انجام گرفت. ابزار جمع اوری اطلاعات دو پرسشنامه شامل پرسشنامه محقق ساخته دارای روایی و پایایی معتبر در چهار بخش اطلاعات جمعیت شناختی، آگاهی، نگرش و عملکرد و نیز پرسشنامه  بهداشت دهان و دندان خود گزارش شده  بود. داده ها با استفاده از نرم افزار SPSS-21 و با کمک آزمونهای توصیفی و تحلیلی T-test ، ANOVA و همبستگی مورد تجزیه و تحلیل قرار گرفتند. یافته ها: میانگین نمره آگاهی دانش آموزان در مورد بهداشت دهان و دندان 07/2± 38/6  از 19 ، میانگین نمره نگرش 35/5± 03/40  از محدوده 50-10 و میانگین نمره عملکرد آنها 9/4± 28/13 از 28 بود. در بررسی عملکرد دانش آموزان، بیشترین رفتاری که آنان انجام می دادند استفاده از خمیر دندان در هنگام مسواک زدن بود در حالیکه کمترین پاسخ مثبت را به استفاده از دهان شویه ضد باکتری داشتند. همچنین میانگین نمره بهداشت دهان و دندان دانش آموزان 19/5± 05/43  از 57 به دست آمد. همبستگی مثبت و معنی داری نیز بین سازه های آگاهی، نگرش، عملکرد و بهداشت دهان و دندان مشاهده گردید. نتیجه گیری: با توجه به  آگاهی ناکافی دانش آموزان مورد بررسی در خصوص بهداشت دهان و دندان و نیز سطح عملکرد پایین آنان بویژه در مواردی از قبیل استفاده از دهان شویه ضدباکتری و حاوی فلوراید، مسواک زدن بعد از هر وعده غذایی و خوردن شیرینی ، ارائه برنامه‌های آموزشی به منظور افزایش آگاهی و عملکرد دانش آموزان بویژه در زمینه های فوق ضروری به نظر می رسد.

Title: بررسی مقایسه ای رابطه رفتار بهداشت دهان با وضعیت سلامت لثه و بهداشت دهان در دانشجویان دندانپزشکی سال اول و آخر

http://tbj.ssu.ac.ir/article-1-2017-fa.html

مقدمه: هدف از انجام این مطالعه ارزیابی تأثیر جنبه های پیشگیری آموزش دندانپزشکی روی رفتار، بهداشت دهان و وضعیت سلامت لثه دانشجویان دندانپزشکی دراستان یزداست. روش بررسی: جهت بررسی رفتار بهداشت دهان با وضعیت لثه وبهداشت دهان دانشجویان دندانپزشکی سال  اول وآخر در دانشگاه علوم پزشکی شهید صدوقی یزد، 51 نفر داوطلب در مطالعه شرکت کرده و پرسشنامهHiroshima University-Dental Behavioral Inventory(HU-DBI) را تکمیل کردند. همچنین معاینه کلینیکی برای اندازه گیری O'leary IndexوGingival Bleeding Indexتوسط یک نفر انجام شد. یافته ها: در این مطالعه پاسخ های دانشجویان سال اول و آخر به سؤالات پرسشنامه HU-DBI مورد مقایسه قرار گرفت و اختلاف معناداری میان دانشجویان دو سال در پاسخ به موارد 1 و 8 و 10 و 15 و 17 و 18 و 20 و 21 مشاهده شد. دانشجویان سال آخر میانگین Gingival  Bleeding  Index و O'leary Index کمتری نسبت به دانشجویان سال اول داشتند. میانگین نمره پرسشنامه HU-DBI در دانشجویان سال آخر بالاتر از دانشجویان سال اول بود. دانشجویان با  نمره پرسشنامه بالاتر، Gingival  Bleeding  Index و O'leary Index کمتری داشتند. نتیجه گیری: این مطالعه نشان می دهد دانشجویان سال آخر رفتار بهداشت دهان، وضعیت سلامت لثه وبهداشت دهان بهتری نسبت به دانشجویان سال اول داشتند و به طور کلی دانشجویان با رفتار بهداشت دهانی بهتر وضعیت سلامت لثه  وبهداشت دهان مطلوب تری داشتند.

Title: پیشگویی کننده‌های سوء مصرف مواد مخدر در دانشجویان: کاربرد تئوری رفتار برنامه ریزی شده

http://tbj.ssu.ac.ir/article-1-2025-fa.html

مقدمه: در ایران سوء مصرف مواد و اعتیاد در حال گسترش است و به طبقه سنی و اجتماعی خاصی محدود نمی‌شود. دانشجویان قشر مستعد و برگزیده جامعه و سازندگان کشور می‌باشند. لذا شناخت عوامل موثر بر پیشگیری از رفتار مصرف مواد مخدر در این افراد ضروری می‌باشد. تئوری رفتار برنامه ریزی شده یکی از مؤثرترین و جامع‌ترین تئورهای تبیین رابطه نگرش و رفتار می‌باشد. این مطالعه با هدف تعیین پیشگویی کننده‌های سوءمصرف مواد مخدر در دانشجویان دانشگاه علوم پزشکی شهید صدوقی یزد با بهره گیری از تئوری رفتار برنامه ریزی شده صورت گرفت. روش بررسی: این پژوهش مقطعی در میان120 نفر از دانشجویان دانشگاه علوم پزشکی شهید صدوقی یزد با روش نمونه گیری تصادفی ساده انجام گرفت. ابزار گردآوری اطلاعات پرسشنامه ای مشتمل بر دو قسمت دموگرافیک و زمینه‌ای با 31 سوال و گویه های مربوط به تئوری رفتار برنامه ریزی شده شامل، نگرش 10 گویه (83/0α=) محدوده نمره 50-10، هنجارهای ذهنی 4 گویه (75/0α=) محدوده نمره 20-4، کنترل رفتار درک شده 2 گویه (81/0α=) محدوده نمره 10-2 و قصد رفتاری 4 گویه (89/0α=) محدوده نمره 20-4 بود. داده‌ها با استفاده از نرم افزار آماری SPSS-18 و با کمک آمار توصیفی و آزمون‌های تحلیلی نظیرتی تست مستقل، آنالیز واریانس یکطرفه، ضریب همبستگی پیرسون و آنالیز رگرسیون خطی در سطح معناداری 05/0 مورد آزمون قرار گرفتند. یافته‌ها: میانگین سن شرکت کنندگان 27/3±49/23  با دامنه 35-18 سال بود. 3/73 درصد زن و 7/26 درصد مرد بودند. 3/8 درصد سابقه مصرف سیگار و 7/6 درصد سابقه مصرف مواد مخدر را داشتند. میانگین نمره نگرش 15/6±8/24، هنجارهای ذهنی52/2±10/11 ، کنترل رفتار درک شده 86/1±85/6 و قصد رفتاری 35/3±95/10 بود. زنان نگرش (016/0=p) و قصد رفتاری (013/0=p) مثبت‌تری در مصرف مواد نسبت به مردان داشتند. متاهلین هنجارهای ذهنی (04/0=p) قصد رفتاری (042/0=p) بالاتری در مصرف مواد نسبت به مجردها داشتند. نگرش، هنجارهای ذهنی و قصد رفتاری با یکدیگر رابطه مستقیم و با کنترل رفتار درک شده نسبت به عدم مصرف مواد رابطه معکوس و معناداری داشتند. نگرش و هنجارهای ذهنی به شکل معناداری به ترتیب 35 و 39 درصد از واریانس قصد رفتاری شرکت کنندگان در خصوص مصرف مواد مخدر را پیشگویی نمودند. نتیجه گیری: نگرش، هنجارهای ذهنی ترغیب کننده، قصد رفتاری مثبت نسبت به مصرف مواد در سطح متوسط و کنترل رفتار درک شده در عدم مصرف مواد در سطح متوسط رو به بالایی در نزد دانشجویان بود. با توجه به اینکه نگرش و هنجارهای ذهنی، پیشگویی کننده‌های مناسبی در قصد رفتاری بودند، لذا، لزوم اجرای برنامه آموزشی متناسب با تئوری، مورد تاکید قرار می‌گیرد.

Title: تجارب بستگان نزدیک بیماران دارای عوامل خطرسازسرطان معده و کارکنان بهداشتی درمانی از تعیین کننده های رفتار تغذیه ای: یک آنالیز محتوای کیفی تئوری محور

http://tbj.ssu.ac.ir/article-1-2028-fa.html

مقدمه:یکی از شایع ترین علل مرگ در گروه های سنی بالای 50سال، سرطان ها هستند که اصلاح سبک زندگی، نقش مهمی در پیشگیری از آنها دارد.رژیم های غذایی، مهمترین عامل در ابتلاء به سرطان معده هستند. هدف پژوهش حاضر، تبیین تجارب بستگان نزدیک بیماران دارای عوامل خطرساز سرطان معده و کارکنان بهداشتی درمانی دانشگاه از تعیین کننده های رفتار تغذیه ای براساس تئوری انگیزش محافظت بود. روش­بررسی:پژوهش کیفی حاضر از نوع تحلیل محتوا به مدت هشت ماه در سال 1392 درمراکز بهداشتی درمانی شهر بابل تحت پوشش داشگاه علوم پزشکی بابل انجام شد. جمع آوری  داده ها به روش مصاحبه نیمه سازمان یافته از 9 نفر از بستگان نزدیک بیماران مبتلا به خطرسازسرطان معده و 19 نفر از کارکنان بهداشتی درمانی دانشگاه انجام شد. تجزیه و تحلیل داده ها همزمان با جمع آوری آنها با استفاده از روش آنالیز محتوای تئوری محور(هدایت شده یا مستقیم) صورت گرفت. یافته­ها:از تجزیه و تحلیل داده ها 487 کد اولیه  و پس از ادغام،186 کد اصلی استخراج شد. این کدها در دو طبقه و هفت زیرطبقه از قبل تعیین شده تئوری انگیزش محافظت (حساسیت درک شده، شدت درک شده، پاداش، ترس، کارآمدی پاسخ درک شده، خودکارآمدی و هزینه- پاسخ درک شده) جایگذاری شدند. اصلی ترین مسئله درک شده ، سطح پایین ترس از بیماری بود. نتیجه­گیری: یافته های حاصل از پژوهش حاضر، نشان گر عوامل تعیین کننده ی موثر بر رفتار تغذیه ای می باشند که می توانند به سیاستگزاران بهداشتی درمانی یاری دهند تا مناسب ترین راهکارها و راهبردها را با هدف تغییر این تعیین کننده ها در جهت کاهش اتخاذ رفتارهای پرخطر تغذیه ای مرتبط با سرطان معده، تهیه وتدوین نمایند.

Title: بررسی رفتارهای پیشگیری کننده از عفونت گوش میانی بر اساس الگوی اعتقاد بهداشتی (HBM) در مادران دارای کودک کمتر از 2 سال درشهریزد

http://tbj.ssu.ac.ir/article-1-2030-fa.html

مقدمه : عفونت گوش میانی یکی از شایع ترین عفونت ها درکودکان است. بیشترین زمان احتمال بروزآن درسنین زیر2سال است.از جمله عوارض حاصل از عفونت گوش میانی: بروزمشکلات شنوایی،آسیب بلندمدت و برگشت ناپذیر به گوش میانی وحلزون ،کاهش بهره هوشی و مشکلات گفتاری می باشد.هدف این مطالعه بررسی رفتارهای پیشگیری کننده ازعفونت گوش میانی براساس الگوی اعتقاد بهداشتی در مادران با کودک کمتر از دو سال، در شهر یزد بود. روش­بررسی : مطالعه حاضر در سال 93-1392 ، به صورت توصیفی مقطعی بر روی 132 نفر از مادران مراجعه کننده به مراکز بهداشتی درمانی شهر یزد  که کودک زیر 2 سال داشته اند ، به صورت تصادفی  با انتخاب 6 مرکز از بین 15 مرکز بهداشتی درمانی  و انتخاب 22 نفر از هر مرکز،انجام شد.داده ها توسط پرسشنامه و به صورت تصادفی جمع آوری شده و با استفاده از نرم افزارSPSS مورد تجزیه و تحلیل قرار گرفت .ازآزمون Tزوجی برای بررسی معنی داری ارتباط بین نمرات سازه ها با متغیرهای دموگرافیک استفاده شد وضریب همبستگی بین نمرات سازه ها توسط آزمون اسپیرمن تعیین گردید. یافته ها: نتایج نشان داد که نمره آگاهی تنها در 5/4% موارد وراهنمایی عمل تنها در1/9%  مواردمطلوب بود. شدت درک شده،موانع درک شده و منافع درک شده به ترتیب در4/92% ، 1/81%  و 7/94% موارد مطلوب  گزارش گردید. بین منافع با خودکارآمدی، حساسیت، شدت، رفتار و آگاهی همبستگی مستقیم وجود داشت.بر اساس یافته ها بین شدت با موانع ،رفتار و حساسیت و خودکارآمدی با موانع و رفتار،همبستگی مستقیم وجود داشت.از طرفی رابطه معنا داری بین سن مادر با شدت ومنافع وبین تعداد فرزند با آگاهی، مشاهده شد. بین سابقه ابتلای کودک به عفونت گوش با راهنمایی عمل، همبستگی معکوس معناداری مشاهده گردید. نتیجه­گیری : بر اساس یافته های این پژوهش  با در نظرگیری نمرات حاصل از سازه ها و پایین بودن نمره آگاهی مادران و از طرفی به دلیل شیوع  بالای عفونت گوش در کودکان زیر2 سال ،اجرای برنامه های آموزشی به منظورافزایش آگاهی مادران و بهبود رفتارهای پیشگیری کننده از عفونت گوش میانی، موثر واقع گردید.

Title: ارتباط اختلال خوردن با افسردگی در دانشجویان پزشکی دانشگاه آزاد اسلامی واحد یزد سال 1392

http://tbj.ssu.ac.ir/article-1-2015-fa.html

مقدمه : اختلال خوردن یکی از اختلالات شایع روانی به شمار می‌رود که باعث مشکلات متعددی در سلامت جسمی و عملکرد روانی و کیفیت زندگی می‌شوند . این مطالعه به منظور بررسی اختلال خوردن و رابطه  آن با افسردگی در دانشجویان پزشکی دانشگاه آزاد یزد در سال 1392 صورت گرفت. روش بررسی: در یک مطالعه توصیفی با استفاده از نمونه گیری در دسترس تعداد 400  نفراز دانشجویان با استفاده از 2 پرسشنامه : نگرش خوردن eat-26 و افسردگی بک، مورد بررسی قرار گرفتند . اطلاعات  بدست آمده با استفاده از آزمون  chi square و fisher exact و Anova مورد تجزیه و تحلیل قرار گرفت. یافته ها: بر اساس نتایج این پژوهش، 5/7 %دانشجویان اختلال خوردن، 8/25 %افسردگی متوسط و 22% افسردگی شدید داشتند  فراوانی نسبی اختلالات خوردن در دانشجویان پزشکی بر حسب سن ،جنس، وضعیت سکونت و وضعیت تاهل یکسان بود.  همچنین بین فراوانی نسبی اختلالات خوردن در دانشجویان با شدت افسردگی رابطه معنی دار وجود داشت .( p=0.01) نتیجه گیری: یافته های مطالعه نشان دادکه بین اختلال خوردن وشدت افسردگی رابطه وجود دارد .بنابراین آموزش دانشجویان توسط مراکز مشاوره در رابطه با اختلالات خوردن وافسردگی می تواند در تشخیص و درمان زودرس این اختلالات موثر باشد

Title: بررسی ارتباط بین مصرف برنج و دفع ادراری آفلاتوکسین M1 در زنان مراجعه کننده به مراکز بهداشتی شهر یزد

http://tbj.ssu.ac.ir/article-1-2018-fa.html

مقدمه: آفلاتوکسین ها  معمولا توسط گونه های آسپرژیلوس فلاووس و آسپرژیلوس پارازیتیکوس تولید می گردد، این سموم در انواع مواد غذایی از جمله برنج یافت می شوند،  هدف از انجام این پژوهش بررسی ارتباط بین مصرف برنج و دفع ادراری آفلاتوکسین  M1 در زنان مراحعه کننده به مراکز بهداشتی شهر یزد می باشد . روش بررسی: این مطالعه از نوع توصیفی – مقطعی بوده  که در سال 1393 با مشارکت 85 نفر از  خانم های ساکن شهر یزد مراجعه کننده به مراکز بهداشتی انجام شد.  با استفاده از پرسشنامه در خصوص مصرف برنج در 72 ساعت قبل سوال شد و غلظت آفلاتوکسین M1 در نمونه های ادرار به روش الایزا اندازگیری شد. یافته ها: نتایج نشان داد  که از مجموع 85 نفر شرکت کننده، 94 درصد از افراد، دفع کننده آفلاتوکسین M1 بودند .  بر اساس آنالیز آماری اختلاف معناداری بین میزان دفع آفلاتوکسین M1 و مصرف برنج (0.015 =p) و همچنین میزان دفع آفلاتوکسین M1 با نوع برنج مصرفی (ایرانی،  وارداتی، مخلوط) (0.023 =p) وجود داشت . نتیجه گیری: مطابق نتایج بدست آمده می توان نتیجه گرفت با افزایش میزان مصرف برنج، دریافت روزانه آفلاتوکسین به طور مستقیم افزایش می یابد. لذا میزان آفلاتوکسین در برنج های مصرفی توسط زنان شهر یزد در حد بالایی بوده و افراد زیادی در معرض غلظت بالایی از افلاتوکسین و خطرات مرتبط با آن قرار دارند.

Title: بررسی عوامل خطر حاملگی خارج رحمی در شهر یزد: مطالعه مورد شاهدی

http://tbj.ssu.ac.ir/article-1-2037-fa.html

مقدمه: حاملگی خارج رحمی یک مشکل بهداشت عمومی بوده و بسیاری از عوامل خطر  آن شناخته نشده اند. این مطالعه با هدف شناسایی عوامل خطر حاملگی خارج رحمی جهت تشخیص و درمان بهتراین بیماری انجام شد. روش­بررسی:این مطالعه مورد شاهدی بر روی 195 مورد حاملگی خارج رحمی (گروه مورد) و 197 نفر حاملگی طبیعی و سزارین (گروه کنترل) در بیمارستان های شهید صدوقی و مادر شهر یزد انجام گردید. گروه کنترل شامل زنان بارداری بودندکه بلافاصله قبل و بعد از افراد گروه مورد برای زایمان مراجعه می کردند. یافته ها: میانگین سنی زنان 6/5±2/28 (حداقل 14 و حداکثر 45) بوده و سن ازدواج در آنان از حداقل 12 تا حداکثر 33 سال متفاوت بود. افراد  دو گروه از نظر شاخص توده بدنی (BMI) تفاوت معنی داری نداشتند (72/0P=). نتایج آزمون کای دو نشان میدهدکه سابقه نازایی(001/0P= 2/5-9/1  CI 95%  18/3OR:)،  سابقه ابتلا به عفونت (01/0P= 53/2-10/1 CI 95% 67/1OR:) ، استفاده از وسیله داخل رحمی (IUD)(03/0P= 16/7-08/1CI95%  78/2OR:) و استعمال دخانیات (01/0P= 21/7-21/1 CI: 95% 96/2OR=) از عوامل خطر حاملگی خارج رحمی بودند. نتیجه­گیری: علت دقیق حاملگی خارج رحمی هنوز کاملا شناخته شده نیست و پاتوژنز حاملگی خارج رحمی به صورت به چندین عامل خطر باقی مانده است. نتایج این مطالعه نشان میدهدکه سابقه نازایی ، استفاده از IUD ، ابتلا به عفونت و استعمال دخانیات، با حاملگی خارج رحمی مرتبط می باشد.

Title: بررسی ارتباط عوامل اقتصادی اجتماعی و باروری با سرطان پستان: مطالعه مورد – شاهدی

http://tbj.ssu.ac.ir/article-1-2020-fa.html

مقدمه: سرطان یکی از سه علت اصلی مرگ در دنیا محسوب می شود. حدود 70% از مرگ هایی که به علت سرطان اتفاق می افتد در کشورهای با وضع اقتصادی اجتماعی پایین اتفاق می افتد. در ایران نیز سرطان پستان اولین سرطان شایع در زنان می باشد واطلاعات موجود حاکی از افزایش بروز سالانه سرطان پستان در زنان ایرانی وکاهش سن ابتلا به این بیماری می باشد. لذا این مطالعه با هدف تعیین ارتباط عوامل اقتصادی اجتماعی و باروری بر سرطان پستان انجام گردید روش­بررسی: این پژوهش به صورت مورد شاهدی مبتنی بر بیمارستان بر روی 150 فرد مبتلا به سرطان پستان و 150 فرد سالم انجام گرفت.  نمونه گیری به روش آسان و جمع آوری اطلاعات به صورت مصاحبه با افراد شرکت کننده انجام گرفت. در نهایت اطلاعات به دست آمده وارد نرم افزار SPSS و جهت آنالیز از آماره های توصیفی و تحلیلی از جمله مجذور کای، نسبت های شانس با حدود اطمینان 95% و نیز رگرسیون لجستیک استفاده شد. یافته­ها: در آنالیز اولیه عوامل تحصیلات، نوع شغل افراد، شغل همسر، درآمد ماهانه، سابقه ضایعه پستانی، شاخص توده بدنی(BMI)، نسبت دور کمر به باسن (WHR) در رابطه با سرطان پستان معنی دار شد و بعد از وارد کردن متغیر های معنی دار در مدل رگرسیون لجستیک چندگانه متغیرهای  درآمد (92/3OR=) و BMI (30BMI>=) (84/3OR=) معنی دار باقی ماندند و سن در اولین تولد زنده (04/5OR=) نیز معنی دار شد. نتیجه گیری: با توجه به اینکه در این مطالعه درآمد پایین، تاخیر در تولد اولین فرزند و چاقی، از عوامل خطرناک و مهم ابتلا به  سرطان پستان بودند، توصیه می شود افرادی که دارای این عوامل خطر هستند نسبت به دریافت مراقبت های لازم جهت پیشگیری و تشخیص زودرس اقدام نمایند.

Title: بررسی رابطه بین سرمایه فرهنگی با بروز انواع رفتارهای پر خطر در بین دانشجویان دانشگاه یزد

http://tbj.ssu.ac.ir/article-1-1046-fa.html

چکیده سابقه و اهداف : دانشجویان در یک جامعه، قشر وسیعی از جمعیت فعال یک کشور را تشکیل می‌دهند، که عوامل متعددی می‌تواند سلامت اجتماعی و روانی آن‌ها را به خطر اندازد و در معرض انواع آسیب‌های اجتماعی و خطرپذیری قرار دهد.هدف مطالعه حاضر بررسی رابطه بین سرمایه اجتماعی و ابعاد سه گانه آن (سرمایه فرهنگی تجسم یافته، سرمایه فرهنگی عینیت یافته و سرمایه فرهنگی نهادینه شده)، از نظر بوردیو، با میزان رفتارهای پر خطر (شامل گرایش به مواد مخدر، سیگار، مشروبات الکلی، رابطه با جنس مخالف و رانندگی خطرناک) در بین دانشجویان دانشگاه یزد می‌باشد. روش بررسی : مطالعه حاضر، مطالعه‌ای میدانی و توصیفی از نوع همبستگی است. ابزار مورد استفاده، پرسشنامه‌های سرمایه فرهنگی و رفتارهای پرخطر می‌باشد و دانشجویان به شیوه نمونه‌گیری طبقه‌بندی متناسب انتخاب گردیدند.پژوهش برروی 384 نفر از دانشجویان مقطع کارشناسی دانشگاه یزد انجام شده است.این افراد به شیوه ی طبقه بندی متناسب با حجم جامعه و به صورت تصادفی انتخاب شده اند.اطلاعات جمع آوری شده با استفاده از نرم افزارAmos ، SPSS 19 مورد تحلیل قرار گرفته است. یافته ها : یافته‌های تحقیق نشان داد بین میزان سرمایه فرهنگی و بروز انواع رفتارهای پر خطر دانشجویان دانشگاه یزد رابطه معنی‌دار منفی وجود دارد و از بین ابعاد سه‌گانه سرمایه فرهنگی، بین سرمایه فرهنگی عینیت تجسم یافته و نهادینه شده و میزان بروز رفتارهای پر خطر، رابطه معنی‌دار منفی وجود دارد؛ و میزان بروز رفتارهای پر خطر در بین پسران، بیش از دختران بوده است. همچنین بین میزان سرمایه فرهنگی و بروز انواع رفتارهای پر خطر به جز میزان گرایش به خشونت، رابطه معنی‌دار منفی وجود داشت.نتایج نشان داد با بالا رفتن سطح فرهنگ و سرمایه فرهنگی جامعه، بخصوص میزان سرمایه فرهنگی تجسم یافته و نهادینه شده از طریق افزایش سطح مطالعه، استفاده از اینترنت و آشنایی به زبان‌های خارجی؛ همچنین تلاش بدست آوردن مدارک تحصیلی، فنی و حرفه‌ای، گواهی‌نامه‌های فرهنگی و هنری و ... می‌توان امیدوار بود که میزان خطرپذیری جوانان و نوجوانان جامعه کاهش پیدا کند و این مسئله می‌تواند برای مسئولین مربوطه راهگشا باشد.

Title: بررسی تأثیر آموزشی مبتنی بر عوامل روانشناختی بر سلامت روان پرستاران مبتلا به کمردرد مزمن: مطالعه کارآزمایی بالینی

http://tbj.ssu.ac.ir/article-1-2023-fa.html

مقدمه: کمردرد مزمن عامل اصلی بروز ناخوشی در شغل پرستاری است. مطالعات مرتبط با سلامت روان پرستاران در محیط کار و تأثیر آن بر کمردرد محدود است، لذا این مطالعه با هدف تعیین تأثیر آموزشی مبتنی بر عوامل روانشناختی بر سلامت روان پرستاران مبتلا به کمردرد مزمن انجام می شود. روش بررسی: پژوهش حاضر مطالعه ای از نوع نیمه تجربی شاهد دار می باشد. جامعه آماری این پژوهش شامل 84 نفر از پرستاران و بهیاران مبتلا به کمردرد مزمن شاغل در بیمارستان ولی عصر (عج) تهران است. افراد به صورت تصادفی انتخاب و به دو گروه شاهد و مورد که هر کدام 42 نفر بودند، تقسیم شدند. برای جمع آوری اطلاعات از سه مقیاس و ابزار استفاده شد. آزمودنیها پرسشنامه دموگرافیک، سنجش درد و بعد سلامت روان پرسشنامه کیفیت زندگی را تکمیل نمودند و سه ماه بعد از مداخله آموزشی مجدد از نظر درد و سلامت روان مورد بررسی قرار گرفتند. داده ها از طریق آمار توصیفی و تحلیلی تجزیه و تحلیل شد.   یافته ها: تعداد افراد در هر دو گروه مورد و شاهد 42 نفر و میانگین سنی آنها به ترتیب 32 و 5/31 سال بود. دو گروه از نظر متغیرهای مورد مطالعه اختلاف معنی داری نداشتند (  5% P > ).  میانگین بعد نشاط پس از مداخله در گروه مورد، از 64/64 به 26/77، بعد سلامت روان، از 28/70 به 95/80، بعد محدودیت نقش بدلیل مشکلات روانی، از 28/64 به 30/87 و بعد عملکرد اجتماعی، از 64/69 به 11/85 افزایش یافت که نشان دهنده تأثیر معنی دار آموزش می باشد  (0001/0 P<  ).       نتیجه گیری: یافته های این مطالعه نشان داد که مداخله آموزشی مبتنی بر عوامل روانشناختی کمردرد، موجب افزایش سلامت روان پرستاران و کاهش کمردرد در آنها می شود. بنابراین با طراحی برنامه آموزشی برای کنترل عوامل روانشناختی در محیط کار می توان از ایجاد یا تشدید کمردرد مزمن در پرستاران جلوگیری کرد.

Title: بررسی آگاهی و نگرش و عملکرد دانش آموزان دختر مقطع متوسطه شهر یزد نسبت به سرطان پوست و رفتارهای محفاظت کننده در برابر نورخورشید

http://tbj.ssu.ac.ir/article-1-2024-fa.html

مقدمه: شایع‌ترین سرطان در اکثر کشورهای دنیا سرطان پوست است و مهم‌ترین عامل خطر آن مواجهه با نور خورشید است. با توجه به لزوم انجام برنامه‌های پیشگیری در دوران کودکی و نوجوانی، این مطالعه با هدف تعیین آگاهی و نگرش و عملکرد دانش‌آموزان دختر مقطع متوسطه شهر یزد نسبت به سرطان پوست و رفتارهای محافظت‌کننده در برابر نورخورشید انجام شد. روش بررسی: این پژوهش از نوع توصیفی مقطعی بوده و بر روی 513 نفر از دانش آموزان دختر مقطع متوسطه شهر یزد که به روش نمونه‌گیری خوشه‌ای چند مرحله‌ای انتخاب شدند انجام شد. ابزار جمع‌آوری داده‌ها پرسشنامه بودکه پایایی آن در مطالعه مظلومی و همکاران با آلفا کرونباخ 81/0 تائید شده بود. داده‌ها با استفاده از نرم افزار SPSS نسخه 5/11 و توسط آمارهای توصیفی و تحلیلی آنالیز شد. یافته‌ها: میانگین سنی شرکت‌کنندگان04/1 ±88/15 بود. میانگین و انحراف‌معیار نمره آگاهی‌، نگرش‌، عملکرد به ترتیب 44/5 ±19/12 (نمره کل=37)، 26/2 ±98/18 (نمره کل=24) و 51/2 ±47/7 (نمره کل=16) بود. شایع‌ترین رفتار محافظت‌کننده از نور خورشید استفاده از ضد‌آفتاب بود (5/71%). بین آگاهی و متغیرهای نگرش (000/0, p=350/0r=)، عملکرد (000/0, p=264/0r=) و سن (010/0, p=134/0r=) همبستگی معنی‌داری مشاهده شد. نتیجه‌گیری: کم بودن آگاهی و مطلوب نبودن رفتارهای محافظت‌کننده از نور خورشید به غیر از استفاده از کرم ضد‌آفتاب در دانش‌آموزان مطالعه حاضر از نیاز برای مداخلات آموزشی مدرسه محور حمایت می‌کند.

Title: بررسی اثر رژیم غذایی کم کالری با و بدون مکمل یاری ویتامین D بر فاکتورهای تن سنجی و عملکرد کبد در بیماران مبتلا به کبد چرب غیر الکلی

http://tbj.ssu.ac.ir/article-1-2031-fa.html

مقدمه: شواهدی مبنی بر اثر ویتامین D برچاقی وجود دارد. همچنین، ارتباط بین کمبود ویتامین D با کبد چرب غیر الکلی در دست بررسی است. هدف این مطالعه، بررسی اثر مکمل یاری ویتامین D در کنار رژیم غذایی کم کالری بر آنزیم های کبدی، درجه درگیری کبد و شاخص های تن سنجی در افراد مبتلا به کبد چرب بود. روش بررسی: این مطالعه یک کارآزمایی بالینی دوسوکور تصادفی می باشد و با مشارکت 80 بیمار مبتلا به کبد چرب غیر الکلی و البته با کمبود ویتامین D انجام گرفت. به افراد جهت همسان سازی برنامه غذایی، رژیم غذایی کم کالری داده شد. هر دو گروه به مدت 12 هفته و هر هفته یک عدد پرل ویتامین D یا دارونما مصرف کردند. آنزیم های کبدی، سونوگرافی کبد و مجاری صفراوی و فاکتورهای تن سنجی در ابتدا و انتهای مطالعه اندازه گیری شد. یافته ها: مطالعه با حضور 73 نفر به اتمام رسید. پس از 12 هفته، آنزیم های کبدی و درجه درگیری کبد در گروه آزمون در مقایسه با گروه دارونما بطور معنی داری کاهش یافت (0.005> P). در گروه آزمون میزان وزن حدود 9 کیلوگرم نسبت به ابتدای مداخله کاهش یافت (0.005> P) . نتیجه گیری: مکمل یاری ویتامین D در کنار رژیم غذایی کم کالری در بیماران مبتلا به کبد چرب غیر الکلی و هایپوویتامینوز D اثر بسزایی در کاهش فاکتورهای تن سنجی و بهبود آنزیم ها و درجه درگیری کبد دارد.

Title: بررسی بافت های غیرمجاز در گوشت چرخ کرده عرضه شده در شهر یزد با روش بافت شناسی

http://tbj.ssu.ac.ir/article-1-2034-fa.html

مقدمه: امروزه مصرف گوشت و آلایش­های دامی در اکثر جوامع بشری رو به افزایش است. با توجه به ارزش اقتصادی گوشت، احتمال استفاده از بافت های غیرمجاز در گوشت چرخ کرده توسط افراد سودجو وجود دارد. بر این اساس مطالعه حاضر به منظور شناسایی بافت های غیرمجاز در گوشت چرخ کرده انجام گرفت. روش بررسی: در این تحقیق تعداد20 نمونه گوشت چرخ کرده منجمد تجاری از فروشگاه های مختلف شهر یزد خریداری شد. سپس هر نمونه به 3 برش مساوی تقسیم شده و از هر قسمت یک قطعه انتخاب شد. نمونه ها در محلول فرمالین 10% بافر تثبیت شدند و آماده سازی معمول بافت ها برای مطالعه میکروسکوپی صورت گرفت و قالب های پارافینی تهیه شد. قالب های پارافینی به ضخامت 6 میکرومتر برش داده شدند و با استفاده از هماتوکسیلین- ائوزین رنگ آمیزی برای مطالعه بافت شناسی صورت گرفت. یافته­ها: در نمونه های مورد مطالعه، بافت عضلانی مخطط بخوبی قابل مشاهده بوده و علاوه برآن ، بافت همبندی ، بافت چربی و بافت عضله صاف نیز مشاهده شد اما در هیچ یک از نمونه ها بافت غیرمجاز تشخیص داده نشد. تنها در یک نمونه ضایعات پوستی کمی مشاهده گردید و در نمونه دیگر بافت استخوان وجود داشت و همچنین در حدود نیمی از نمونه ها ضایعات امعاء و احشا مشهود بود و مقاطعی از لیگامنت مشاهده شد. نتیجه گیری: تحقیق حاضر نشان داده گوشت مورد استفاده در اکثر این نمونه ها برای تهیه این فرآورده، از کیفیت خوبی برخوردار نبوده که با توجه به اهمیت بهداشتی و کیفی گوشت به کار رفته ، روش بافت شناسی برای شناسایی تقلبات در فرآورده های گوشتی پیشنهاد می شود.

Title: اثر پرتوالکترونی برخواص فیزیکی وشیمیایی و ویژگی های حسی بستنی سنتی

http://tbj.ssu.ac.ir/article-1-2036-fa.html

مقدمه: با توجه به افزایش کاربرد فناوری پرتودهی در ارتقای ایمنی محصولات غذایی نیاز است که اثرات این فناوری جدید بر خواص ظاهری و ویژگی های  حسی محصولات مختلف مورد ارزیابی قرار گیرد به این دلیل که اولین عاملی که توجه مصرف کننده را برای خرید یک محصول غذایی جلب می کند، ویژگیهای ظاهری و خواص حسی آن است. هدف از این مطالعه تعیین اثر پرتوالکترونی بر خواص فیزیکوشیمیایی و ویژگی های حسی بستنی سنتی می باشد. روش­بررسی: نمونه های بستنی سنتی زعفرانی از فروشگاهی در سطح شهر خریداری و جهت منجمد نگه داشتن نمونه، نمونه ها درون جعبه سرد قرار داده شده و به آزمایشگاه منتقل شدند .نمونه ها با حفظ شرایط انجماد تحت تابش پرتوالکترونی با دزهای 1،2،3 و5 کیلوگرمی قرار گرفتند و پس از یک هفته نگهداری در دمای°C 18 - ،آزمون های فیزیکوشیمیایی شامل رطوبت،چربی، قند و pH و آزمون های حسی شامل رنگ، بو، مزه و پذیرش کلی بر روی آن  انجام شد. یافته­ها: نتایج نشان دادند که تفاوت قابل ملاحظه ای بین نمونه های گروه تیمار (پرتودهی شده) و گروه شاهد از نظر میزان قند، چربی و pH وجود نداشت. اما اختلاف معنا داری در میزان رطوبت اندازه گیری شده در نمونه های تیمار شده با پرتو الکترونی و نمونه شاهد دیده شد (05/0p<). همچنین  اگر چه دز های 1 و 2 کیلوگری اثر معنی داری روی ویژگی های حسی محصول نداشت، اما با افزایش دز پرتودهی به مقادیر بالاتر از 2 کیلو گری ،پذیرش کلی و رنگ به طور قابل توجهی کاهش یافت (05/0p<). نتیجه­گیری: نتایج مطالعه حاضر نشان می دهد که حداکثر دز قابل توصیه برای پرتودهی محصول بستنی سنتی 2 کیلوگری است و دز های بالاتر موجب افت کیفیت محصول می شود.

Title: اثر فرایند کشک سازی صنعتی بر روی باقیمانده آفلاتوکسینM1

http://tbj.ssu.ac.ir/article-1-2038-fa.html

مقدمه:آفلاتوکسینM1ترکیبی بسیار سمی بوده که طی فرایندو نگهداری شیر پایدار است. از این رو در زمره یک آلاینده شیر و فراورده های لبنی است که می تواند عوارض خطرناک برای انسان را به دنبال داشته باشد.در این زمینه مطالعات متعددی کارایی اثر فرایند در فراورده های لبنیاتی  بر روی حذف آفلاتوکسین M1را نشان داده اند.بنابراین هدف از انجام این تحقیق، مطالعه اثر فرایند تهیه  کشک صنعتی بر روی کاهش آفلاتوکسینM1 اضافه شده بطور مصنوعی در فرایند طبیعی کشک سازی می باشد. روش­بررسی:در این مطالعه کشک صنعتی از شیر گاو(شیر بدون چربی)که در یک سطح به مقدار25/0میکروگرم در لیتر آفلاتوکسین M1 به صورت مصنوعی آلوده شده بود در 3 تکرار، تهیه شد و تاثیر فرایند کشک سازی صنعتی بر روی آفلاتوکسین M1   مورد مطالعه قرار گرفت.برای اندازه گیری مقدار آفلاتوکسین  M1از روش کراماتوگرافی مایع با فشار بالا استفاده شد. یافته­ها:.در تولید کشک صنعتی در غلظت های مساوی بین شیر اولیه و کشک صنعتی مقدار کاهش آفلاتوکسین M1 به میزان 91 درصد بود. و این مقدار کاهش آفلاتوکسینM1 بین شیر اولیه و کشک صنعتی از لحاظ آماری در سطح (052/0(P <معنی دار بود. نتیجه­گیری:نتایج این مطالعه نشان میدهد که فرایند کشک سازی صنعتی میتواند خطرات سم آفلاتوکسین M1که انسان به وسیله مصرف لبنیات یا شیر با آن مواجه است را کاهش بدهد

Title: بررسی مقدار آفلاتوکسین M1 در شیر مادران شهر یزد در دو فصل تابستان و زمستان در سال 1392

http://tbj.ssu.ac.ir/article-1-2039-fa.html

مقدمه: آفلاتوکسین ها یکی از قوی ترین سموم تولید شده توسط گونه های مختلفی از کپک آسپرژیلوس هستند که موجب سرکوب سیستم ایمنی، سرطانزایی، موتاژنی و ناقص الخلقه زایی می شوند. هدف اصلی از انجام این پایان نامه بررسی مقدار آفلاتوکسین M1 در دو فصل تابستان و زمستان در شیر مادران شهر یزد می باشد. روش بررسی: این مطالعه از نوع مقطعی می باشد. در سال 1392 طی دو فصل تابستان و زمستان نمونه های شیراز بین 80 مادر شیرده مراجعه کننده به مراکز بهداشتی درمانی شهر یزد انتخاب گردید. برای اندازه گیری میزان آفلاتوکسین M1 از روش الایزا استفاده شد. یافته ها: : از مجموع تعداد 80 نمونه، تعداد 6 مورد(5/7درصد) آلوده به آفلاتوکسین M1 بودند. میزان آلودگی در فصل زمستان به طور معنی داری بیشتر از فصل تابستان بود(05/0p<)، اما رابطه معناداری بین میزان این سم در نمونه های صبح و عصر مشاهده نشد (05/0p>). نتیجه گیری: با توجه به یافته های حاصل از این تحقیق می توان نتیجه گرفت که آموزش بانوان شهر یزد به منظورآشنایی با منابع تولید کننده آفلاتوکسین M1 در شیر ضروری می باشد که می تواند راهی در جهت کاهش عوارض این سم  باشد.

Title: بررسی جذب آفلاتوکسین B1 توسط نانوذرات سلولز کونژوگه شده با آپتامر در شرایط اسیدی، بازی و خنثی

http://tbj.ssu.ac.ir/article-1-2040-fa.html

مقدمه: سم آفلاتوکسین باعث سرطان کبد می شود و جذب آن در مواد غذایی بسیار ضروری است. هدف از این مطالعه بررسی جذب آفلاتوکسین B1 توسط نانوذرات سلولز کونژوگه شده با آپتامر در شرایط اسیدی، بازی و خنثی بود. روش­ بررسی: نخست نانوذرات سلولز با روش هیدرولیز اسیدی سنتز و سپس با کمک کراس لینکر به آپتامر اصلاح شده کونژوگه گردید. در مرحله بعد، غلظت‌های سریال نانوذرات کونژوگه شده (mg/mL 6/0 و 25/1 و 5/2 و 5 و 10) بطور جداگانه با محلول آفلاتوکسین با غلظت µg/mL 1000 مخلوط و در دمای ºC 37 و در pH های 1 و 7 و 13  بمدت 5/0 ساعت انکوبه گردیدند. سپس درصد جذب برای هر لوله در طول موج 340 نانومتر محاسبه گردید. یافته­ها: کاهش pH منجر به افزایش جذب تا حدود 40 درصد شد. از نظر آماری، تفاوت معنی داری بین مقدار جذب در شرایط اسیدی با مقدار جذب در شرایط بازی و خنثی دیده شد ( 05/0P< ). نتیجه گیری: آفلاتوکسین به خوبی می‌تواند به نانوذرات کونژوگه شده جذب شود و این قدرت جذب در شرایط اسیدی افزایش می یابد.

Title: طراحی و تهیه نمونه آزمایشگاهی قرص های مکمل پر انرژی جهت ورزشکاران، گردشگران و آسیب دیدگان

http://tbj.ssu.ac.ir/article-1-2041-fa.html

مقدمه: ورزشکاران از جمله کوه نوردان، صحرا نوردان و دریا نوردان و گردشگرانی که در طبیعت دچار سانحه و یا  گم می شوند و نیز افرادی که بلایای طبیعی موجب بی خانمان شدن آنها شده است نیاز به تامین حداقل انرژی از طریق مکمل ها یا قرص های پر انرژی مورد نیاز تا زمان رسیدن کمک های بیشتر را دارند. در این مطالعه هدف،طراحی و ساخت نمونه آزمایشگاهی این گونه قرص های پر انرژی میباشد. روش بررسی:به صورت آزمایشگاهی، 15 فرمولاسیون مختلف طراحی و ساخته شد و با هماهنگی با دانشکده داروسازی دانشگاه علوم پزشکی اصفهان این فرمولاسیون ها به منظور قابلیت تبدیل به قرص های پر کالری با هدف تامین حداقل 20 کیلوکالری به ازای هر قرص وحداقل مقادیر پروتئین روزانه (حدود 25 گرم) و نیز ویتامین ها و مواد معدنی مختلف مورد بررسی و تهیه قرار گرفتند. یافته ها: نمونه آزمایشگاهی قرص های 5 گرمی که هر کدام حاوی 21 کیوکالری انرژی بودند، تهیه شد. بهترین فرمولاسیونی که مشخص شد برای تهیه قرص، مقرون به صرفه و قابل اجرا است شامل 16 درصد روغن زیتون، 36 درصد مالتودکسترین، 25 درصد پودر پروتئین، 17 درصد دانه کنجد خام و 6 درصد جوانه خالص گندم بود. با در نظر گرفتن اینکه هر فرد روزانه 25 عدد از قرص ها را استفاده کند، روزانه 525 کیلوکالری انرژی برای فرد تامین میشود. اگر هر جعبه از قرص ها به طور متوسط 2/1 کیلوگرم طراحی شود، قرص ها می توانند حداقل انرژی مورد نیاز،پروتئین،ویتامین ها و مواد معدنی  را تا 10 روز تامین نمایند.  نتیجه­گیری:  تامین حداقل انرژی و مواد مغذی مورد نیاز برای زنده ماندن  ورزشکاران، گردشگران و آسیب دیدگان از بلایای طبیعی  تا مدت هفت روز  یا حتی بیشتر به دلیل حجم اندک قرص های طراحی شده امکان پذیر می باشد و قابلیت تولید صنعتی در ایران را دارد.

Title: بررسی سطح توسعه‌ یافتگی سلامت شهرستان‌های استان یزد با استفاده از تکنیک تاکسونومی عددی در سال‌ 1392

http://tbj.ssu.ac.ir/article-1-2027-fa.html

مقدمه: از اوایل دهه 90 که مفهوم توسعه انسانی به عنوان یکی از ملاک‌های ارزیابی توسعه یافتگی مطرح شد، بهبود سلامت و بهداشت جامعه که رکن اساسی این توسعه را تشکیل می‌داد، به چالشی برای دولت‌ها مبدل گردید. لذا تحقیق حاضر با هدف تعیین سطح توسعه‌ یافتگی سلامت شهرستان‌های استان یزد با استفاده از تکنیک تاکسونومی عددی در سال‌ 1392، انجام گرفت. روش بررسی: این پژوهش از نوع توصیفی بوده که به بررسی وضعیت شاخص‌های بهداشتی و درمانی در 10 شهرستان استان یزد ، در سال 1392 پرداخته است. داده‌های مورد نیاز بر اساس نظر خبرگان و مراجعه به معاونت‌های توسعه مدیریت و منابع، غذا و دارو، بهداشت و درمان دانشگاه علوم پزشکی شهید صدوقی یزد، مرکز بهداشت استان یزد، مرکز آمار استان یزد و سازمان بهزیستی استان یزد جمع‌آوری گردید و با استفاده از تکنیک‌های تحلیل سلسله مراتبی و تاکسونومی عددی تحلیل گردید. یافته‌ها: شهرستان‌های مهریز و ابرکوه به ترتیب با درجه توسعه یافتگی 474/0 و 987/0، برخوردارترین و محروم‌ترین شهرستان‌های استان می‌باشند و شهرستان‌های بافق، یزد، اردکان، میبد، تفت، بهاباد، صدوق و خاتم به ترتیب در این بین قرار می‌گیرند. نتیجه‌گیری: اختلاف و شکاف در سطح توسعه یافتگی بهداشت و درمان، بین شهرستان‌های استان یزد وجود دارد که امید است مسئولان کشوری و استانی در تخصیص امکانات بهداشتی و درمانی به هر یک از شهرستان‌های استان یزد، میزان برخورداری و توسعه یافتگی هر شهرستان را ملاک عمل قرار داده و بر مبنای آن، برنامه‌ریزی نمایند.

Title: بررسی رابطه بین معنویت سازمانی واعتیاد به کار در پرستاران بیمارستان های آموزشی دانشگاه علوم پزشکی شهید صدوقی یزد در سال 1393

http://tbj.ssu.ac.ir/article-1-2029-fa.html

مقدمه: معنویت در محیط کار، باعث بهبود عملکرد، ایجاد رضایت و انگیزه در کارکنان و افزایش بهره وری سازمان می شود. از طرفی انجام بیش از حد کار که اعتیاد به کار نامیده می شود باعث رسیدن سازمان به اهدافش می شود. هدف پژوهش حاضر بررسی رابطه بین معنویت سازمانی و اعتیاد به کار می باشد. روش بررسی:پژوهش حاضر، از نوع توصیفی و همبستگی بوده و به صورت مقطعی در سال 1393 انجام شد. جامعه پژوهش کلیه پرستاران بیمارستان های آموزشی شهر یزد بودندکه 170 نفر از آنها به روش نمونه گیری طبقه ای و سیستماتیک انتخاب شدند. به منظور گردآوری داده ها از پرسشنامه معنویت سازمانی میلیمن و پرسش نامه  اعتیاد به کار اسپنس و رابینز استفاده شد. داده ها  با استفاده از  نرم افزار SPSS  وآزمون های آماری t-test، کای اسکوئر وضریب همبستگی پیرسون تجزیه و تحلیل گردید . یافته ها: میانگین نمره معنویت سازمانی 72/62 ومیانگین نمره اعتیاد به کار 08/70 به دست آمد.نتایج نشان داد که بین معنویت سازمانی و اعتیاد به کار، رابطه مثبت و معنادار وجود دارد. نتیجه­گیری : براساس یافته های پژوهش،  معنویت سازمانی از متغیرهای تاثیرگذار بر  اعتیاد به کار است.لذا سیاست گذارن سازمان ها می توانند با بهره گیری از معنویت، حداکثر توان کارکنان را در رسیدن به اهداف سازمان به کار گیرند. همچنین راهبردهای مناسب را درجهت توجه به سلامت کارکنانی که اعتیاد به کار مفرط دارند اتخاذ نمایند.

Title: بررسی نگرش کارکنان در زمینه مدیریت کیفیت فراگیر با استفاده از منطق فازی، مطالعه موردی در بیمارستانهای آموزشی شهر یزد

http://tbj.ssu.ac.ir/article-1-2032-fa.html

مقدمه: در مدیریت کیفیت فراگیر، کیفیت تنها یک پدیده تحسین برانگیز نیست بلکه حق مسلم مشتری است و از طریق درگیری و مشارکت کلیه کارکنان، مدیران و مشتریان یک سازمان ایجاد خواهد شد. این مطالعه به منظور بررسی نگرش کارکنان بیمارستان­های آموزشی شهر یزد در زمینه مدیریت کیفیت فراگیر با استفاده از منطق فازی، طراحی شده است. روش بررسی: پژوهش حاضر از نوع توصیفی تحلیلی می­باشد که به صورت مقطعی انجام گرفته است.جامعه مورد مطالعه شامل کلیه کارکنان بیمارستان­های آموزشی شهر یزد بود که از بین آن­ها تعداد 235 نفر به صورت تصادفی طبقه­ای انتخاب و مورد مطالعه قرار گرفتند. اطلاعات مورد نیاز از طریق پرسشنامه  جمع­آوری شد. از آنجا که تئوری فازی، در مقابل تئوری منطقی، رویکردی مناسب‌تری برای سنجش متغیرهای کلامی است، بنابراین این مقاله با بکارگیری منطق فازی ، نگرش کارکنان را در زمینه مدیریت کیفیت فراگیر تعیین می­کند. یافته­ها: یافته­ها حاکی از آن بود که ابعاد "شناسایی و آموزش کارکنان"، "توانمند­سازی و کار تیمی کارکنان" و "حمایت و رهبری مدیریت عالی سازمان" به ترتیب در رتبه­های اول، دوم و سوم اهمیت از نظر کارکنان برخوردار هستند. نتیجه­گیری: معیارهای شناسایی و آموزش کارکنان و کارتیمی و حمایت و رهبری مدیریت عالی سازمان برانگیزند­ه­های مهم مدیریت کیفیت فراگیر محسوب می­شود. بنابراین در برنامه­های ارتقای مدیریت کیفیت جامع، این ابعاد را با توجه به درجه اهمیت باید در اولویت قرار داد و در جهت فراتر رفتن کیفیت خدمات تلاش نمود.

Title: بررسی ارتباط وزن گیری بارداری و وزن هنگام تولد نوزاد در شهر یزد در سال 1392

http://tbj.ssu.ac.ir/article-1-2033-fa.html

مقدمه : عوامل متنوعی روی سلامت مادر و جنین تاثیر می گذارند که تغذیه مناسب مادر یکی از آنها است. وزن گیری مادر در بارداری یکی از معیارهای معتبر برای ارزیابی وضعیت تغذیه است.  هدف از انجام این مطالعه بررسی ارتباط وزن گیری دوران بارداری و وزن هنگام تولد در شهر یزد است. روش بررسی: در این پژوهش مقطعی ، اطلاعات پرونده 200  مادر باردار 40-16 ساله مراجعه کننده به مراکز بهداشتی درمانی یزد مورد بررسی قرار گرفت. نوزادان با وزن کمتر از 2500 گرم LBW و با وزن بیشتر از 2500 گرم نرمال درنظر گرفته شدند. برای آنالیز داده ها از 16 SPSS  استفاده شد. یافته ها: میانگین سن  زنان در این مطالعه 65/26 سال بود. میانگین کلی وزن گیری بارداری مادران 39/12 کیلوگرم بود. میانگین وزن هنگام تولد نوزادان 3147 کیلوگرم  بود که از این میان 5/7 درصد  (15 نفر) LBW و 5/92 درصد (185 نفر) وزن نرمال داشتند. میانگین BMI قبل از بارداری 08/24 کیلوگرم بر متر مربع بدست آمد. همبستگی میان وزن گیری مادران طی بارداری و وزن هنگام تولد نوزادان معنی دار نبود. نتیجه گیری: از آنجا که وزن هنگام تولد نوزاد ممکن است تحت تاثیر عوامل دیگری مانند سلامت عمومی و روانی مادر و همچنین مرتبه بارداری قرار گیرد ، میتوان با شناسایی این عوامل مراقب بیشتری از مادران باردار به عمل آورد.

Title: تحلیل شاخص های شهر سالم در شهر کرمان در سال 1391

http://tbj.ssu.ac.ir/article-1-2035-fa.html

مقدمه : شهرنشینی گسترده و سریع موجب شده که امروزه سلامت افراد به مخاطره افتاده و موجب مطرح شدن بحث سلامت در مجامع عمومی و عملی کردن راهکارهایی از جمله اجرای پروژه شهر سالم شود.هدف این مطالعه ارزیابی شاخصهای شهر سالم (بهداشتی-زیست محیطی و اجتماعی-اقتصادی) در شهر کرمان در سال 1390 می باشد. روش­بررسی :روش این تحقیق ترکیبی(کیفی-کمی) می باشد که جهت روش کیفی از روش اسنادی و پیمایشی(توزیع پرسشنامه)استفاده شده است. جامعه آماری ساکنین شهر کرمان بودند همچنین برای سنجش میزان مشارکت افراد در مباحث سلامت از روش کمی و از نرم افزار SPSS و آزمون های آماری همچون ضریب همبستگی،تحلیل واریانس یکطرفه و t  نمونه های مستقل استفاده شده است. یافته ها:نتایج نشان داد که از 8 شاخص  بهداشتی فقط 3 مورد(پوشش واکسیناسیون کودکان زیر یکسال،تولد نوزادان کم وزن و مزگ و میر کودکان زیر یکسال) با استانداردها منطبق بودند و از 6 شاخص اجتماعی و اقتصادی  2 مورد شاخص کیفیت مسکن و سقط جنین و از 8 شاخص زیست محیطی نیز تنها 2 مورد (کیفیت آب آشامیدنی و کیفیت جمع آوری زباله) با استانداردها مطابقت داشتند.همچنین یافته های پژوهش در خصوص تمایل افراد به  مشارکت در امور مربوط به سلامت فردی و اجتماعی نشان داد که 87% آنها تمایل دارند و در این رابطه جنسیت و سن آنها تاثیری بر میزان مشارکت آنها نداشته اما افراد با تحصیلات بالاتر تمایل بیشتری به مشارکت داشتند. نتیجه­گیری: بطور کلی شهر کرمان از نظر شاخصهای بهداشتی،اجتماعی-اقتصادی و زیست محیطی وضعیت مطلوبی ندارد اما ساکنین این شهر تمایل زیادی به بهبود وضعیت سلامت شخصی و محیط خود دارند.

Title: بررسی ویژگی های شیمیایی و حسی پنیرفراسودمند تهیه شده از مخلوط شیرگاو و شیرسویا

http://tbj.ssu.ac.ir/article-1-2043-fa.html

مقدمه: درسالهای اخیر باتوجه به شیوع اضافه وزن، چاقی و بیماریهای قلبی درجامعه وارتباط آن با افزایش میزان چربی دریافتی،چربی اشباع شده وکلسترول غذا،تولید محصولات غذایی فراسودمند باکاهش سطح کلسترول وچربی اشباع شده جهت سلامت عموم افراد جامعه با استفاده از سویا و فرآورده های حاصل از آن به علت قیمت مناسب، عدم وجودکلسترول و لاکتوز،وجود ترکیبات ایزوفلاونوئیدی،چربی های غیراشباع و ضروری مورد توجه قرار گرفته است. هدف ازاین پژوهش، ارزیابی ویژگی های شیمیایی و حسی پنیر اولیه فراسودمند تهیه شده از نسبت های مختلف مخلوط شیر گاو و شیر سویا می باشد. روش بررسی: این تحقیق یک مطالعه تجربی می باشد که شیر سویا در 5 سطح (0، 5، 10، 15 و 20 %) و کلرید کلسیم با نسبت 4/0 درصد با شیر گاو مخلوط و در تهیه پنیر اولیه بکار رفت. ویژگی های شیمیایی و حسی نمونه ها بر اساس استانداردهای ملی تعیین گردید نتایج در قالب طرح پایه کاملاً تصادفی با آرایش فاکتوریل و توسط نرم افزار آماری 16 Minitabدر معرض تجزیه واریانس قرار گرفت. مقایسه میانگین داده ها بر اساس آزمون چند دامنه ای دانکن انجامشد. یافته ها: نتایج نشان داد که با افزایش جایگزینی شیر سویا ، میزان پروتیین، چربی، ماده خشک ، اسیدهای چرب غیر اشباع ضروری ( اسیدهایاولئیک، لینولئیک و لینولنیک) و امتیاز بافت نمونه های پنیر تولیدی افزایش و میزان کلسترول ، اسیدهای چرب اشباع ، امتیاز رنگ و طعم  نمونه های پنیر تولیدی در مقایسه با نمونه شاهد کاهش یافت. نمونه حاوی شیر گاو 6/84% ، شیر سویا 15% و کلروکلسیم 4/0% در مقایسه با سایر نمونه ها از نظر ویژگی های شیمیایی مناسب تر و خصوصیات حسی آن قابل قبول می باشد. نتیجه گیری:با انتخاب نسبت های مناسب جایگزینی شیر سویا با شیر گاو در تولید پنیر می توان محصولی تولید کرد که دارای ویژگی های کیفی مطلوب و سلامتی بخش برای مصرف کننده باشد و به عنوان غذای فراسودمند در تغذیه افراد جامعه به خصوص سالمندان مورد استفاده قرار گیرد.

Title: بررسی رابطه ی ابعاد رهبری تحول آفرین و درگیری شغلی کارکنان از دیدگاه مدیران دربیمارستانهای آموزشی شهر یزد در سال 1392

http://tbj.ssu.ac.ir/article-1-2044-fa.html

مقدمه: در طلیعه قرن جدید در سازمان های بهداشتی و درمانی، همواره نیاز به تغییرات ریشه ای و خلاقیت های کاری احساس می شود. امروزه سبک  جدیدی از مفهوم رهبری با عنوان رهبری تحول آفرین مطرح است ،که این سبک  سرپرستان کارکنان را تحت تاثیر قرار می دهد تا توانایی های خود را ارتقا دهند و موجب  به بوجود آوردن تغییرات مثبت در عملکرد پرسنل برای انجام بهتر کارها و تقویت یادگیری و افزایش توانایی کارکنان میشوند. از سوی دیگر مفهوم  درگیری شغلی  نیز یکی ازموضوعات مهم سازمانی است که به درجه ای که اشخاص به لحاظ روان شناختی با شغل فعلی شان هویت یابی می کنند، اشاره دارد. هدف این پژوهش بررسی رابطه ی ابعاد رهبری تحول آفرین و درگیری شغلی کارکنان از دیدگاه مدیران  دربیمارستانهای آموزشی دانشگاه علوم پزشکی شهید صدوقی یزد در سال  1392 بود . روش بررسی : پژوهش حاضر از نوع توصیفی –تحلیلی می باشد .جامعه ی پژوهشی شامل کلیه ی مدیران(عالی،میانی،عملیاتی) بیمارستان های آموزشی (شهید صدوقی ،رهنمون ،افشار،سوانح و سوختگی) بودندکه به علت محدود بودن جامعه ی پژوهش کل جامعه به عنوان نمونه انتخاب شد( نفر180 )داده ها توسط پرسشنامه ی رهبری تحول آفرین از دیدگاه مدیران باس و الیو و نیز پرسشنامه ی درگیری شغلی توماس جمع آوری  و با استفاده از شاخص های  آماری توصیفی  چون میانگین ،انحراف معیار و ضریب همبستگی پیرسون ودر قالب نرم افزار spss18   تحلیل شد. یافته ها: یافته ها نشان می دهند که بین رهبری تحول آفرین وچهار بعد آن (ترغیب ذهنی،انگیزش الهام بخش ،نفوذ آرمانی ،ملاحظات فردی) با درگیری شغلی در چهار بیمارستان باr=289 وp=0/01 رابطه ی مثبت و معناداری وجود دارد. نتیجه گیری: رهبر تحول افرین در بیمارستانها موجب به بوجود آوردن تغییرات مثبت در عملکرد پرسنل برای انجام بهتر کارها و تقویت یادگیری و افزایش توانایی کارکنان میشود.
